# Improved pharmacodynamics of epidermal growth factor via microneedles-based self-powered transcutaneous electrical stimulation

**DOI:** 10.1038/s41467-022-34716-5

**Published:** 2022-11-14

**Authors:** Yuan Yang, Ruizeng Luo, Shengyu Chao, Jiangtao Xue, Dongjie Jiang, Yun Hao Feng, Xin Dong Guo, Dan Luo, Jiaping Zhang, Zhou Li, Zhong Lin Wang

**Affiliations:** 1 Beijing Institute of Nanoenergy and Nanosystems, Chinese Academy of Sciences, Beijing, 101400 China; 2 Department of Plastic Surgery, State Key Laboratory of Trauma, Burns and Combined Injury, Southwest Hospital, Third Military Medical University (Army Medical University), Chongqing, 400038 China; 3 School of Nanoscience and Technology, University of Chinese Academy of Sciences, Beijing, 100049 China; 4 Institute of Engineering Medicine, Beijing Institute of Technology, Beijing, 100081 China; 5 Beijing Laboratory of Biomedical Materials, College of Materials Science and Engineering, Beijing University of Chemical Technology, Beijing, 100029 China; 6 Center of Nanoenergy Research, School of Physical Science and Technology, Guangxi University, Nanning, 530004 China; 7 Institute for Stem Cell and Regeneration, Chinese Academy of Sciences, Beijing, 100101 China; 8 Georgia Institute of Technology, Atlanta, GA 30332 0245 USA

**Keywords:** Biomedical engineering, Bionanoelectronics, Drug delivery, Protein delivery

## Abstract

Epidermal growth factor is an excellent drug for promoting wound healing; however, its conventional administration strategies are associated with pharmacodynamic challenges, such as low transdermal permeability, reduction, and receptor desensitization. Here, we develop a microneedle-based self-powered transcutaneous electrical stimulation system (mn-STESS) by integrating a sliding free-standing triboelectric nanogenerator with a microneedle patch to achieve improved epidermal growth factor pharmacodynamics. We show that the mn-STESS facilitates drug penetration and utilization by using microneedles to pierce the stratum corneum. More importantly, we find that it converts the mechanical energy of finger sliding into electricity and mediates transcutaneous electrical stimulation through microneedles. We demonstrate that the electrical stimulation applied by mn-STESS acts as an “adjuvant” that suppresses the reduction of epidermal growth factor by glutathione and upregulates its receptor expression in keratinocyte cells, successfully compensating for receptor desensitization. Collectively, this work highlights the promise of self-powered electrical adjuvants in improving drug pharmacodynamics, creating combinatorial therapeutic strategies for traditional drugs.

## Abstract (web summary)

The use of epidermal growth factor for wound healing is limited by transdermal permeability, reduction, and receptor desensitization. Here the authors develop a microneedle-based self-powered transcutaneous electrical stimulation system to overcome these challenges.

## Introduction

Epidermal growth factor (EGF) is a small polypeptide consisting of 53 amino acid residues and three disulfide bonds, with the latter determining biological activity. EGF plays a significant role in regulating cell growth, survival, migration, apoptosis, proliferation, and differentiation^1,2^. The biological effects of EGF are exerted by binding to EGF receptor (EGFR), which activates Ras/mitogen-activated protein kinase (Ras/MAPK), phosphatidylinositol 3-kinase/AKT (PI3K/AKT) and phospholipase C-ki*γ*/protein after the autophosphorylation of receptor tyrosine kinase (RTK)^3,4^. EGFR is distributed on the surface of fibroblasts, endothelial cells, smooth muscle cells, and epidermal cells^5^. After binding EGF, EGFR signaling promotes cell chemotaxis and remodeling, triggering the formation of granulation tissue and epidermis. Due to its excellent performance in accelerating epidermal regeneration, EGF is commonly used in the treatment of surgical wounds, burns, and diabetic ulcers^6–8^.

EGF administration is associated with several pharmacodynamic challenges. Firstly, regarding topical administration in the form of creams commonly used in clinics, the high molecular weight of EGF (Mw≈6 kDa) limits its penetration of the stratum corneum^9,10^. Thus, only trace amounts of EGF can pass through the hair follicle to the basal layer and influence surviving keratinocytes^11^. Although EGF can be delivered transdermally by injection, this poses a risk of bacterial infection for wound patients, and the pain caused by the injection reduces patient compliance, which is undesirable for wound treatment^12,13^. Secondly, EGF has low stability in vivo; this is because glutathione (GSH) breaks the disulfide bonds that stabilize the EGF structure, resulting in the reduction and inactivation of EGF, which greatly reduces its efficacy^14,15^. Finally, EGFR has high affinity for EGF, and their specific binding promotes cell proliferation and migration by activating the downstream factor PI3K via the EGF/EGFR pathway^16^. However, EGF can cause rapid endocytosis of EGFR into endosomes and eventual degradation in lysosomes^17^. Thus, long-term EGF treatment leads to desensitization and attenuation of EGFR, terminating the signaling pathway^18,19^. Therefore, improving pharmacodynamics of EGF in wound healing can be approached from the following aspects: (i) increase the penetration rate in a minimally invasive manner; (ii) maintain chemical stability of EGF and prevent its reduction by GSH; (iii) upregulate EGFR expression to compensate for receptor desensitization.

It has been proved that physiological electric fields could upregulate the expression of growth factor receptors in cardiomyocytes and corneal epithelial cells^20–22^. This inspired us to develop a device with transcutaneous electrical stimulation (ES) and transdermal drug delivery capabilities to improve drug permeability while compensating for receptor desensitization. Advanced microneedle is an ideal therapeutic medium. As minimally invasive transdermal devices, microneedles (MNs; length <1 mm) can penetrate the stratum corneum without bleeding or pain^23–25^. MNs can be fabricated from different materials, such as silicon, glass, polymers, or even metals, endowing them with good mechanical properties, solubility, adhesion, and electrical conductivity^26–28^. Micromolecular and macromolecular drugs can be encapsulated in dissolvable MNs and diffused directly into the skin as MNs degrades^29,30^. It is worth mentioning that conductive MNs could be used as electrodes to reach the low-resistance dermis (~10 kΩ) and bypass the high-resistance stratum corneum (~10 MΩ), thus enabling transcutaneous ES to improve the pharmacodynamics of EGF.

In this study, we designed a microneedle-based self-powered transcutaneous electrical stimulation system (mn-STESS) to improve the pharmacodynamics of EGF in terms of wound healing. The integrated mn-STESS consisted of a sliding free-standing triboelectric nanogenerator (sf-TENG) and two-stage gold coated polylactic acid/cross-linked gelatin–cross-linked hyaluronic acid (PLA-Au/cGel-cHA) composite microneedle patches (CMNPs). The device was wireless, passive, and easily attached to the skin. The sf-TENG converted the biomechanical energy generated by finger sliding into biosafe microcurrent without causing skin damage or drug inactivation. CMNP penetrated the stratum corneum and continuously released EGF into the skin for 24 h. Meanwhile, CMNP utilized the current generated by the sf-TENG for transcutaneous ES. As an electrical adjuvant, self-powered ES suppressed the GSH-mediated reduction of EGF by regulating the motility of both molecules, thus maintaining the stability of exogenous EGF. Long-term cell and animal experiments also showed that ES simultaneously upregulated EGFR expression to compensate for receptor desensitization. The constructed mn-STESS could effectively improve the pharmacodynamics of EGF to aid wound healing.

## Result and discussion

### Design and integration of the microneedles-based self-powered transcutaneous electrical stimulation system

**Fig. 1:**
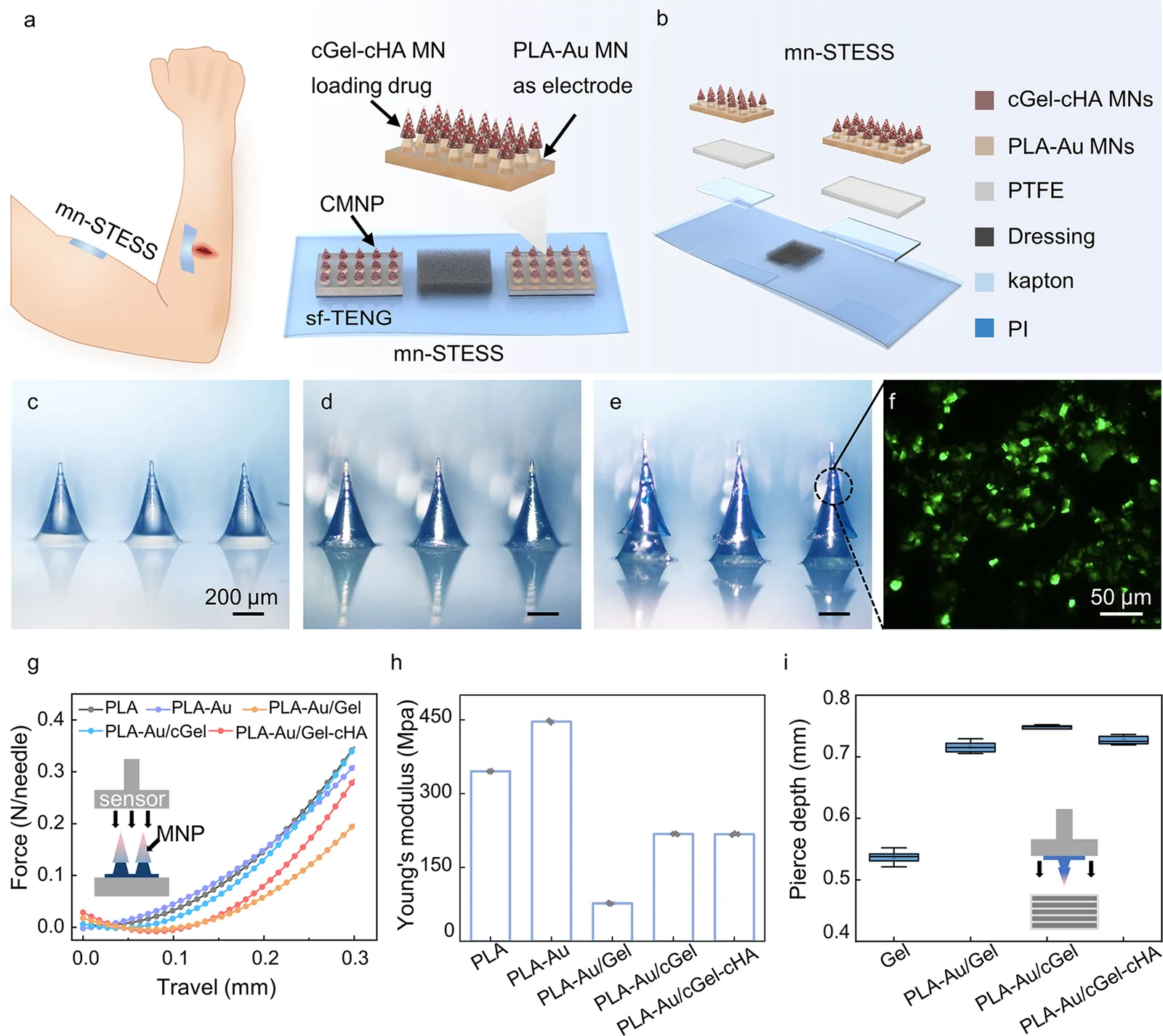
Overview of the mn-STESS. **a** The mn-STESS consisted of sf-TENG, CMNP, and dressing. The CMNP composed of cGel-cHA MNs loading drug and PLA-Au MNs as electrode. **b** Three-dimensional structure of the mn-STESS. **c**–**e** Brightfield micrographs of PLA MNP, PLA-Au MNP, and CMNP. **f** cHA microparticles encapsulated in CMNP. **g** Typical force-displacement curve of the compression force of MNs; (inset) schematic of the experimental setup. **h** Young’s moduli of MNs. (*n* = 3 independent samples. Data are presented as mean ± SEM). **i** Parafilm penetration depths of MNs; (inset) schematic of the experimental setup. (*n* = 5 independent samples. Data are presented as center line, limits and whiskers, 25%–75%). Source data are provided as a Source Data file.

The mn-STESS was designed based on sf-TENG and two-stage CMNPs to improve the pharmacodynamics of EGF in wound healing and could adhere to the skin (Fig. 1a). The sf-TENG was composed of triboelectric, dielectric and electrode layers. Polyimide (PI) film was used as the triboelectric layer and polytetrafluoroethylene (PTFE) thin films covered with Kapton tape served as the dielectric layers (Fig. 1b). Polylactic acid-coated gold microelectrode array patches (PLA-Au MNP) were utilized as the electrodes. The drug-loaded cross-linked gelatin and cross-linked hyaluronic acid microneedles (cGel-cHA MNs) were covered on the PLA-Au MNP to create a two-stage CMNP. After applying CMNP to the skin, the drug loaded in the cGel-cHA MNs and current generated by sf-TENG were simultaneously introduced into the skin^31–33^. A chitosan dressing was placed in the middle of mn-STESS to absorb the wound exudate and cushion the vertical force exerted by finger sliding^34^.

### Fabrication and characterization of two stage CMNP

The two-stage CMNP was fabricated as follows (Supplementary Fig. 1). Due to the excellent mechanical properties, good biocompatibility and biodegradability of polylactic acid (PLA)^35,36^, PLA microneedle patches (PLA MNPs) were first fabricated by a thermoforming process (5 × 5 MN array on a 7 × 7 mm patch). Considering that the shape, height, base diameter and array density of the microneedles will affect their puncture performance; in this work, the microneedles were designed to be conical with a height of 550 μm, a base width of 300 μm and the inter-needle spacing of 500 μm (Fig. 1c). A PLA-Au MNP was subsequently generated by sputtering a 50 nm-thick layer of gold on the surface of the PLA MNP (Fig. 1d). Finally, drug-loaded cGel-cHA MNs with the length of 550 μm were prepared by vacuuming and covered on the tip of PLA-Au MNs to fabricate the two-stage PLA-Au/cGel-cHA CMNP. The total height of the two-stage needle body was 750 μm, with an overlapping needle length of ~350 μm (Fig. 1e and Supplementary Fig. 2). After penetration of the dermis, the PLA-Au MNP could introduce current generated by sf-TENG to form transcutaneous ES and directly deliver the drug into the skin by cGel-cHA MNs.

As a water-soluble macromolecule, the absorption of EGF through the skin stratum corneum is problematic^37^. EGF could be delivered into skin using CMNP fabricated from biosafe cross-linked gelatin (cGel) and cross-linked hyaluronic acid (cHA) microparticles. In this study, Gel and HA were cross-linked by genipin and 1,4-butanediol diglycidyl ether (BDDE), respectively, to control the EGF release rate and mechanical properties of CMNP. Genipin was nucleophilically attacked by the primary amine group of the Gel molecule, resulting in opening of the dihydropyran ring to form a heterocyclic amine (Supplementary Fig. 3a). Gel molecules formed a network structure with genipin as a cross-linking bridge. As shown in the Fourier transform-infrared (FT-IR) spectra (Supplementary Fig. 3b), the characteristic peaks of Gel at 1245, 1540, and 1650 cm^−1^ were assigned to the C = O bonds of carbonyl groups, N–H bonds of the amino groups, and N–H bonds of amide III groups, respectively. In the cGel sample, the intensity of the N–H band decreased, and the peak shifted slightly in the low wavenumber direction (from 1245 to 1240 cm^−1^), indicating that genipin and amino groups of Gel undergo a cross-linking reaction^38,39^. For cHA microparticles, there was a new peak at 1445 cm^−1^ in the FT-IR spectrum, attributing to the ether bonds (C–O–C) formed by the cross-linking of BDDE epoxy groups and HA hydroxyl groups (Supplementary Fig. 4a, b)^40^. The diameter of the cHA microparticles was about 15 μm (Supplementary Fig. 4c) and the diameter increased to ~20 μm after encapsulation of EGF (Fig. 1f), because it swelled by absorbing the EGF solution. In our study, EGF was directly encapsulated in cGel and cHA microparticles, and thus did not participate in the chemical cross-linking process of the hydrogel. HPLC and ELISA results further confirmed that the structure and activity of EGF did not change after encapsulation by cGel and cHA hydrogels (Supplementary Fig. 5a, b).

The mechanical properties of MNs were crucial for their transdermal ability. Conical-shaped PLA MNs have suitable geometry (height, base width and array density) and sufficient mechanical strength to pierce the skin^41–43^. The ability of the two-stage MN to penetrate the skin mainly depended on the second-stage drug-loaded MN located at the tip. The force-displacement curves presented the force applied to the MN gradually increased with displacement. The slope of the PLA-Au/cGel MNs was greater than that of PLA-Au/Gel MNs because the dense network structure formed by cross-linked molecular chains improved the mechanical properties of Gel. The slope of PLA-Au/cGel-cHA MNs was shallower than that of PLA-Au/cGel MNs, possibly due to the incorporation of cHA microparticles in the MNs matrix (Fig. 1g and Supplementary Fig. 6a, b). The Young’s modulus of the PLA-Au/cGel-cHA MNs was ~100 MPa, which was markedly higher than that of skin (~0.13 MPa)^44^, this indicated that CMNP (with conical-shaped PLA-Au/cGel-cHA MNs) with sufficient mechanical properties had the potential to penetrate the skin tissue (Fig. 1h). The penetration depth of MNs was evaluated by using them to pierce Parafilm that mimicked the skin. According to the holes formed in each membrane, the MN penetration depth was calculated from the number of layers pierced. The thickness of the human epidermis and dermis is ~200 μm and 2 mm, respectively. Compared with the one-stage Gel MNs, the two-stage MNs had a greater penetration depth (730 μm) (Fig. 1i), confirming that CMNP could well penetrate and be completely deposited in the dermal tissue, facilitating drug delivery into the skin. PLA-Au/cGel-cHA MNs were further applied to both the porcine skin and living mouse skin to further confirm their ability to penetrate the skin. As expected, application of microneedles created indelible puncture sites on the skin surface and in the penetration cavities. The histological section images of skin tissues with H&E staining showed that CMNP could easily pierce the epidermis and completely penetrate the drug-loaded needle into the dermis (Supplementary Fig. 7a–f).

### Working principle and output properties of sf-TENG

**Fig. 2:**
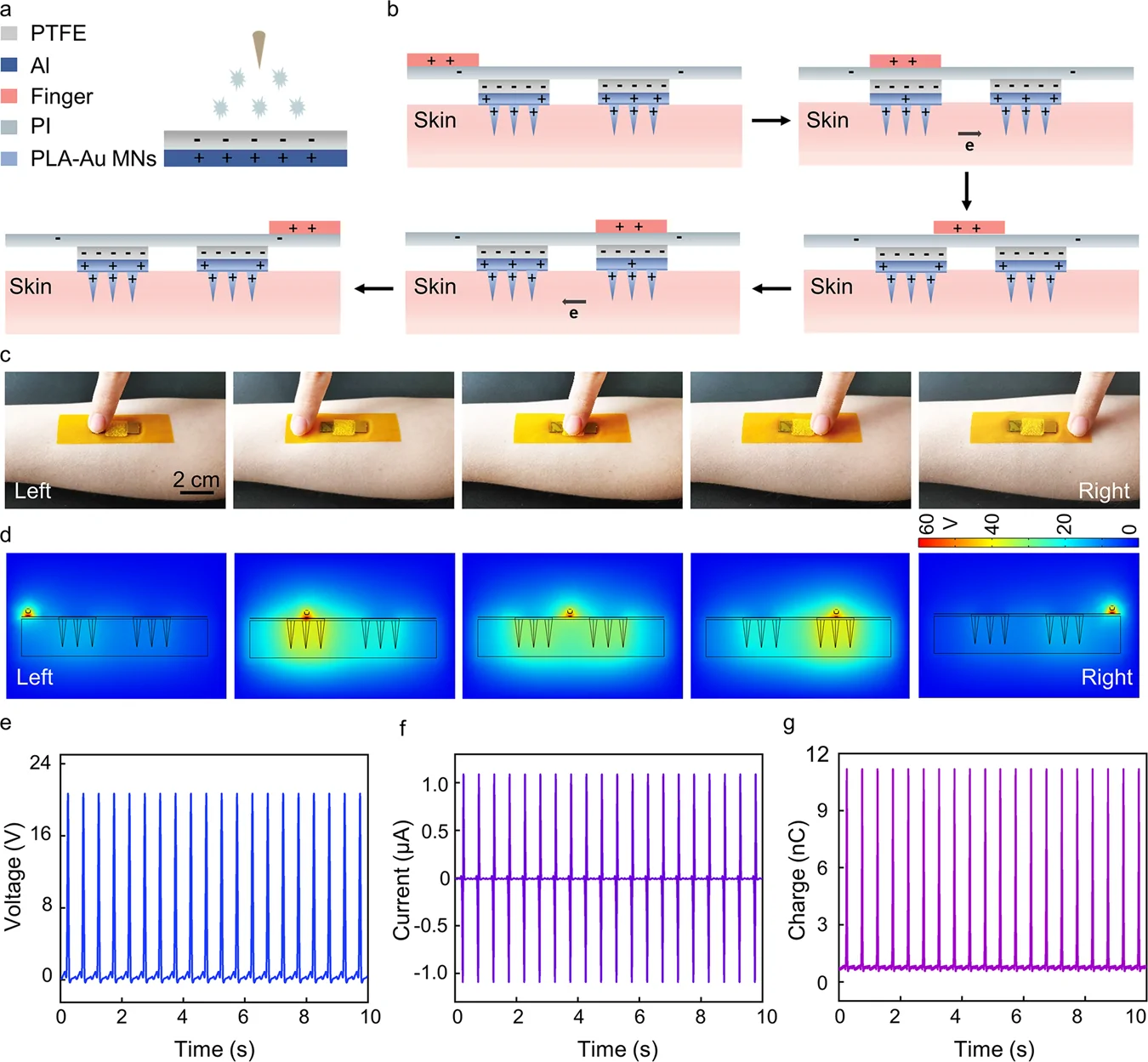
Electric performance of mn-STESS. **a** Sketch of a corona discharge system. **b** Schematic of the working principle of sf-TENG. **c**, **d** Photographs and COMSOL simulation schematics of finger sliding from left to right on sf-TENG. **e**–**g** V_OC_, I_SC_, and Q_SC_ of the sf-TENG. Source data are provided as a Source Data file.

The corona discharge method was used to increase the surface charge density of the PTFE dielectric layer (Fig. 2a), thereby enhancing the output of sf-TENG^45^. The working principle of sf-TENG was shown in Fig. 2b. The finger and outermost PI were used as the triboelectric layers. Due to electrostatic induction, the surface of the finger would carry positive charge when in contact with the outermost PI, and the charge distribution of the PLA-Au MNP (electrode layers) changed when the finger slid on the PI. As indicated by the COMSOL simulation, when the finger slid from left to right on the sf-TENG, the potential of the right PLA-Au MNP was higher than that of the left, driving electron flow from the left PLA-Au electrode to the right electrode through the skin as an external load (Fig. 2c, d). Similarly, when the finger moved to the left, electrons flowed in the opposite direction. The output performances of sf-TENG under different achievable finger sliding frequencies were investigated, where the sliding frequency of 2 Hz had the most suitable output parameters (Supplementary Fig. 8a). In our experiments, a pigskin-wrapped mechanical linear motor was used instead of finger stroking to drive the sf-TENG, and the resulting open-circuit voltage (V_OC_), short-circuit current (I_SC_), and short-circuit transferred charge (Q_SC_) were about 20 V, 1 μA, and 11 nC, respectively (Fig. 2e–g), which were consistent with the output performance of sf-TENG driven by human finger sliding (Supplementary Fig. 8b–d and Supplementary Movie 1).

### mn-STESS improved permeability and utilization of EGF

**Fig. 3:**
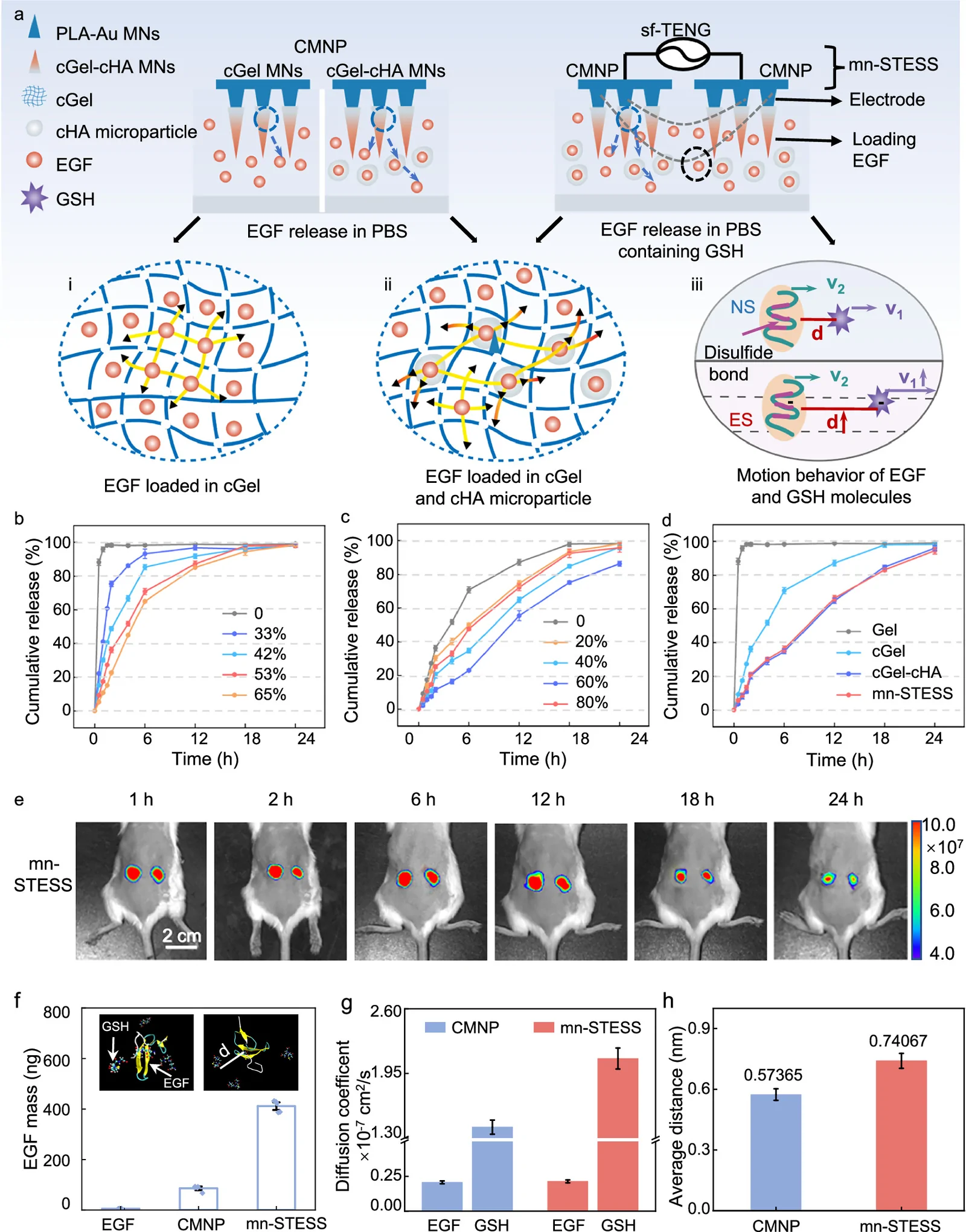
Drug delivery in vitro and in vivo. **a** Schematic of EGF release from MNP based on different drug-loaded materials: (i) cGel MNs; (ii) cGel-cHA MNs in CMNP and mn-STESS; (iii) motion of EGF and GSH molecules in the CMNP (NS) and mn-STESS (ES) groups. **b** EGF release efficiency from the cGel MNs with the different crosslinking degree. (*n* = 3 independent samples. Data are presented as mean ± SEM). c EGF release efficiency from cGel-cHA MNs with the different cHA microparticle contents. (*n* = 3 independent samples. Data are presented as mean ± SEM). **d** EGF release efficiency from Gel MNs, cGel MNs, cGel-cHA MNs, and mn-STESS. (*n* = 3 independent samples, mean ± SEM). **e** Fluorescence images of skin penetrated by mn-STESS at different times. Fluorescence intensities are indicated by a color scale (right). Blue to red presents the minimum to maximum fluorescence intensity. **f** Mass of EGF from CMNP and mn-STESS in GSH solution; (inset) motion behavior of EGF and GSH in the CMNP (NS, left) and mn-STESS (ES, right) groups; the yellow parts indicate the disulfide bond of EGF. (*n* = 5 independent samples. Data are presented as mean ± SEM). **g** Diffusion coefficients of EGF and GSH under NS and ES. Data are presented as mean ± SEM, and the errors are generated by the linear fit of the mean square displacement from simulation trajectory analysis. **h** Distance between EGF and GSH molecules under ES; CMNP was set as control group. Data are presented as mean ± SEM, and the errors are generated by the linear fit of the mean square displacement from simulation trajectory analysis. Source data are provided as a Source Data file.

As a drug carrier, the second-stage cGel-cHA MN had a tightly connected polymer network controlling drug release in a physicochemical manner^46^. To enable continuous daily treatment, the cross-linking degree of cGel and cHA particle content could be adjusted to control the sustained release time of CMNP to 24 h. The effect of cGel MNs cross-linking degree on EGF release kinetics was first explored in vitro. EGF was released from cGel MNs due to water absorption and swelling of cGel (Fig. 3a(i)). The release rate of cGel MNs mainly depended on the degree of cross-linking of cGel, which increased with the volume of genipin solution (Supplementary Fig. 9a). After cross-linking, the Gel solution changed from yellow to dark blue (Supplementary Fig. 9b). The EGF release rate decreased with increasing cGel MNs cross-linking (Fig. 3b and Supplementary Table 1) and the sustained release time also increased, which was attributed to the formation of a tighter network structure of cGel with a higher degree of cross-linking. Both 53% and 65% cross-linked cGel MNs could continuously release drug for a long period (90% release in 18 h) (Supplementary Fig. 10a). However, the poor flowability of cGel with 65% cross-linking hampered the preparation of MNs (Supplementary Fig. 9c). Therefore, cGel with a cross-linking degree of 53% was used to fabricate MNs for subsequent studies.

Sustained drug release by MNs could be further prolonged by introducing cHA particles into cGel. As the drug release process of cGel-cHA MNs, the outer layer of cGel swelled upon contact with solution, allowing the encapsulated drug and drug-loaded cHA particles to slowly exude (Fig. 3a(ii)). Then, the escaped cHA particles further swelled and slowly released the drug. Different cHA contents exhibited differentiated drug release kinetics: as the content of cHA microparticles in cGel MNs increased, more EGF was introduced into cHA microparticles to slow down the EGF release; however, when the cHA content exceeded 60%, MNs released drug more rapidly due to lack of cGel encapsulation (Fig. 3c and Supplementary Table 2). MNs with 40% and 60% cHA microparticle contents enabled sustained drug release over 24 h (Supplementary Fig. 10b). Moreover, the higher proportion of cHA microparticle also influenced the mechanical performance of MNs by causing discontinuities in the distribution of each component. At a cHA microparticle content of 60%, the parafilm penetration rate of cGel-cHA MNs decreased rapidly, indicating that the mechanical properties of cGel-cHA MNs deteriorated (Supplementary Fig. 10c). Taken together, considering the optimal mechanical and drug release kinetic properties of MNs, the optimized drug-loaded portion of the CMNP was composed of 53% cross-linked cGel and 40% cHA microparticles.

The effect of the electric field generated by sf-TENG on the movement behavior of drug molecules was further investigated^47^. The in vitro drug release kinetics indicated no difference in drug release rate between mn-STESS and CMNP (Fig. 3d and Supplementary Table 3); moreover, currents at different sliding frequencies in the mn-STESS also did not affect the drug release rate (Supplementary Fig. 10d). The mn-STESS maintained sustained release of EGF for 24 h (Supplementary Fig. 10e), implying that the current generated by mn-STESS hardly modulated the physicochemical process of microneedle-based drug release. The penetration depth and release kinetics of the drug were visualized in vitro in skin-mimicking hydrogels prepared with 15% porcine gelatin. The results showed that the penetration depth of fluorescently labeled EGF delivered by the mn-STESS was ~990 μm, while the drugs administered by conventional dressings only stayed on the surface. Similarly, the current generated by mn-STESS under different sliding frequencies also did not affect the penetration depth of drug (Supplementary Fig. 11a, b). The release kinetics of mn-STESS was further studied in vivo. Material toxicity tests showed that all components of the CMNP were highly biocompatible (Supplementary Fig. 12a, b). Two CMNPs loaded with FITC-labeled EGF were applied to the back skin of mice and the drug release process was visualized using an in vivo imaging system (IVIS) (Fig. 3e and Supplementary Fig. 13a, b). The imaging results showed that the fluorescence area and intensity at the administration site gradually increased during the initial 12 h, indicating that the drug was continuously released from the CMNP and diffused in the skin. Subsequently, the fluorescence area and intensity decreased simultaneously, as the encapsulated drugs gradually diffused into the deep tissues and were absorbed into the systemic circulation^48^. Consistent with the drug release results in vitro, mn-STESS continuously released drug over 24 h in vivo. Therefore, mn-STESS could improve the pharmacodynamics by enhancing EGF penetration without changing the drug release rate.

### mn-STESS maintained the stability of EGF by suppressing GSH reduction

Reduced GSH (M_w_ ≈ 307.33), a highly active antioxidant, is widely distributed in the skin tissue fluid with the concentration of 2~20 μM^49,50^. GSH could reduce EGF by opening the disulfide bond, rendering it inactive^51,52^. mn-STESS enhanced EGF activity by suppressing the reduction of GSH. ELISA confirmed that although the ES produced by mn-STESS had little effect on the concentrations of monocomponent EGF and GSH (Supplementary Fig. 14a, b). Interestingly, when both components were present, the EGF concentration was 4.8 times higher in the mn-STESS group compared to the CMNP group (Fig. 3f), confirming the inhibitory effect of ES on GSH reduction. The underlying mechanism might be attributed to the regulation of molecular motion by the electric field (Fig. 3a(iii)). A molecular dynamics simulation showed that the ES generated by mn-STESS had little effect on the movement rate of EGF, consistent with the drug release results for the mn-STESS group described above. However, the diffusion coefficient of GSH in the mn-STESS group was 1.5 times that of the CMNP group, resulting in a significant increase in the intermolecular distance between EGF and GSH (Fig. 3g, h and Supplementary Fig. 14c), thereby reducing the collision probability between GSH and EGF and preventing destruction of the EGF disulfide bond by GSH.

### mn-STESS upregulated EGFR expression in HaCaT cells to compensate for receptor desensitization

**Fig. 4:**
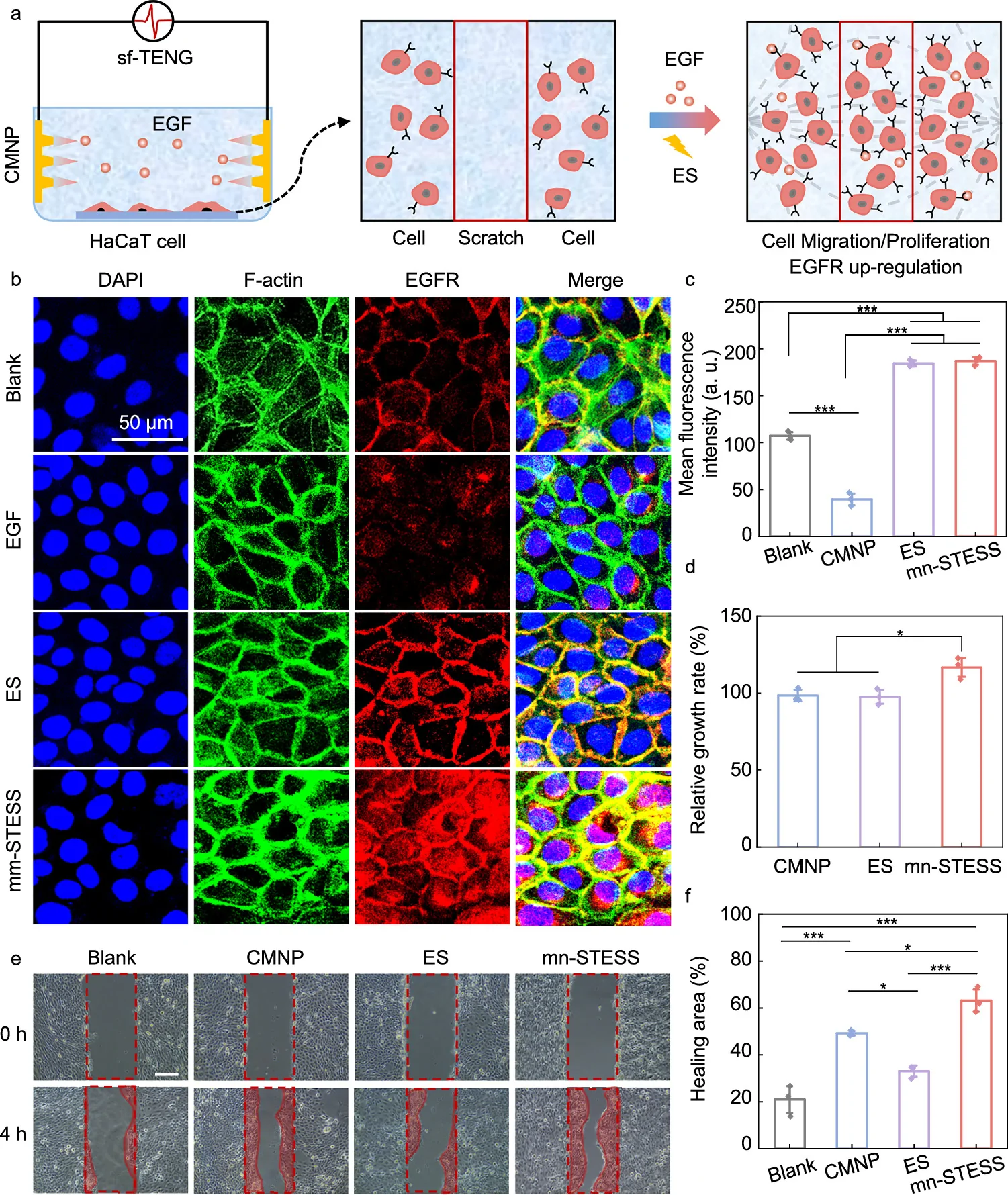
Effects of electrical stimulation synergistically with EGF on HaCaT cell behavior. **a** mn-STESS generated ES and released EGF to promote cell proliferation and migration. **b** Representative fluorescence images of F-action (green) and EGFR (red) in HaCaT cells treated with CMNP (EGF), ES, and mn-STESS (EGF and ES); scale bar, 50 µm. **c** Mean fluorescence intensities of EGFR expression in HaCaT cells. (*n* = 3 independent samples. ***p < 0.001. All statistical analyses were performed by one-way ANOVA. Data are presented as mean ± SEM). **d** Relative growth rate of HaCaT cells treated with CMNP (EGF), ES, and mn-STESS (EGF and ES). (*n* = 3 independent samples. *p < 0.05. All statistical analyses were performed by one-way ANOVA. Data are presented as mean ± SEM). **e** Representative images of HaCaT cell migration; red area indicated the migrated cells; scale bar, 200 µm. **f** Migration area of HaCaT cells treated with CMNP (EGF), ES, and mn-STESS (EGF and ES). (*n* = 3 independent samples. *p < 0.05 and ***p < 0.001. All statistical analyses were performed by one-way ANOVA. Data are presented as mean ± SEM). Source data are provided as a Source Data file.

EGFR is a membrane surface receptor that specifically binds to EGF with high affinity^37,53^. However, continuous administration of EGF desensitizes cellular EGFR, resulting in a significant reduction in drug efficacy. During the natural wound healing process, increased EGFR expression in corneal epithelial cells is associated with bioelectric fields, suggesting that ES is a potential strategy to compensate for receptor desensitization. To investigate whether the ES produced by mn-STESS enhanced EGFR expression and improved EGF pharmacodynamics, a wound model was further established in vitro, and EGFR immunofluorescence staining was performed on epidermal cells under different treatment modes (Fig. 4a). The results showed that the expression level of EGFR in the CMNP group was the lowest, which was only 37% of that in the Blank group, confirming that severe receptor desensitization occurred under EGF administration. The EGFR expression was significantly increased under the intervention of ES. Despite the simultaneous presence of ES and EGFR administration in the mn-STESS group, the receptor sensitization induced by self-powered ES overwhelmed the drug-induced downregulation of EGFR, and EGFR expression in the mn-STESS group was 4.7-fold that in the CMNP group (Fig. 4b, c). Therefore, mn-STESS significantly compensated for receptor desensitization, suggesting that it has potential for improving pharmacodynamics.

Activation of the EGF/EGFR signaling pathway could promote cell proliferation^33^. Cell viability tests exhibited that the monotherapy with ES (1 μA current) and EGF administration (10 ng/mL) had no effect on cell proliferation after 24 h. However, the combination of ES and EGF administration in the mn-STESS group accelerated cell proliferation at the same cell culture time (Fig. 4d). Specific binding of EGF and EGFR alters the actin cytoskeletal structure, thereby promoting cell migration^54^. Fluorescent staining confirmed that EGF and mn-STESS significantly promoted F-actin aggregation and altered cytoskeletal distribution in HaCaT cells (Fig. 4b). Moreover, cell scratching experiments mimicking wounds confirmed that mn-STESS further promoted cell migration by improving the pharmacodynamics of EGF. After 4 h of intervention, the percentage of wound healing area in the Blank, CMNP, ES, and mn-STESS groups was ~20.9%, 49.3%, 33.0%, and 63.2%, respectively (Fig. 4e, f). The scratch healing area ratio of the CMNP group was about 2.4-fold that of the Blank group, confirming the effect of EGF on promoting cell migration. The percentage healed area in the ES group was slightly but non-significantly larger than that in the Blank group, likely because the alternating current generated by sf-TENG in mn-STESS was far less effective than direct current in promoting cell migration^55^. The wound healing area in the mn-STESS group was even 28.2% larger than that in the CMNP group. Because the direct effect of alternating current ES on cell migration was weak, this remarkable healing effect was attributed to the improved EGF pharmacodynamics induced by mn-STESS. In addition, mn-STESS did not generate additional thermal perturbations to affect cell migration. As the current of the built-in sf-TENG was only 1 μA, its theoretical thermal energy was only 0.02 J, which hardly caused the temperature of the culture medium to increase (Supplementary Fig. 15).

PI3K is a downstream molecule of the EGF/EGFR signaling pathway^16^. To confirm that cell proliferation and migration were related to activation of the EGF/EGFR signaling pathway, PI3K expression of HaCaT cells in different groups was assessed by immunofluorescence staining. PI3K expression in the ES group did not correspond to the EGFR content due to lower phosphorylation levels of EGFR at limited endogenous EGF concentrations. As shown by the staining results, there was no difference in PI3K positivity between the ES and Blank groups. The addition of exogenous EGF in the CMNP group significantly increased the rate of PI3K positivity. In the mn-STESS group, the PI3K content was further increased under the stimulation of self-powered ES (Supplementary Fig. 16a, b). These results demonstrated that mn-STESS promoted the expression of EGFR through self-powered ES to improve pharmacodynamics, thereby enhancing the binding of EGF to EGFR, promoting the phosphorylation of EGFR, and accelerating cell migration and proliferation by activating the EGFR signaling pathway.

### Improved pharmacodynamics of EGF via mn-STESS in wound healing

A mouse full-thickness skin wound model (with a 7 × 7 mm defect) was further established to evaluate the therapeutic effect of mn-STESS. CMNPs penetrated the skin barrier and directly delivered the drug to the skin (Supplementary Fig. 17a). Hematoxylin and eosin (H&E) staining showed that CMNP penetrated the dermis to a depth of ~700 μm. Moreover, the long-term skin penetration of CMNPs did not trigger inflammation, and the skin returned to a normal state 20 min after CMNP removal (Supplementary Fig. 17b, c). Compared with traditional chitosan dressing loaded with EGF (CD-EGF), CMNPs could better promote wound healing because of high permeability and utilization of EGF (Supplementary Fig. 17d, e). To ensure that the mn-STESS could provide ES to wounds in animal models, the wound potentials and currents before and after mn-STESS intervention were monitored (Supplementary Fig. 18). The peak wound current after mn-STESS intervention was 1 μA, identical to the short-circuit current of mn-STESS. The peak wound potential after mn-STESS intervention was slightly higher than the endogenous wound electrical potential. The wound current and potential waveforms exhibited the same frequency (2 Hz) as sf-TENG. It was worth mentioning that the horizontal sliding of the finger when driving the mn-STESS produced little mechanical stimulation to the wound, which was attributed to the buffering of the stress by the chitosan dressing covering the wound surface. At the same time, animal experiments also confirmed that simple mechanical stimulation by finger moving had no significant effect on wound healing (Supplementary Fig. 19a, b). Furthermore, the PI layer on the surface of mn-STESS also shielded the thermal conduction of finger sliding, and the skin surface temperature remained almost unchanged after driving mn-STESS for 4 h (Supplementary Fig. 20).

**Fig. 5:**
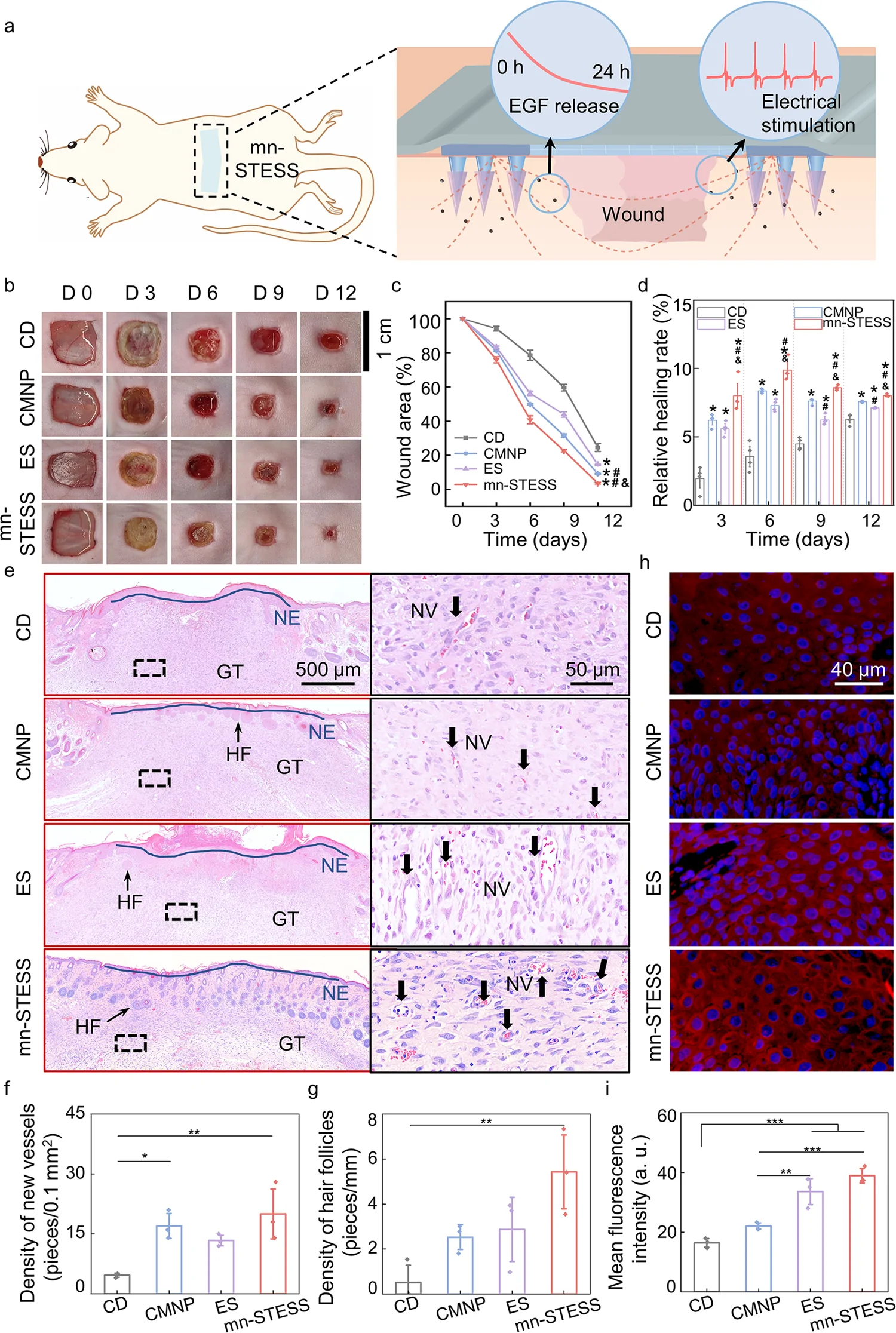
mn-STESS promoted wound healing in vivo. **a** Schematic showing how mn-STESS promoted wound healing. **b** Representative digital images of wound areas treated with CMNP (EGF) and mn-STESS (EGF and ES) on days 0, 3, 6, 9, and 12 (*n* = 6). **c**, **d** Quantitative analysis of wound area and relative healing rate for each group on days 0, 3, 6, 9, and 12. (*n* = 4 independent samples. *p < 0.05. *, # and & indicate the significant differences between other groups and CD, CMNP, ES, respectively. All statistical analyses were performed by one-way ANOVA. Data are presented as mean ± SEM). **e** H&E staining of wound healing tissue showing new epithelium (NE), new granulation tissue (GT), and new hair follicles (HF); the magnified H&E staining in GT showing new vessels (NVs). Blue lines, black rectangles, thin black arrows and thick black arrows indicate NE, GT, HF, and NV, respectively. **f** Quantitative statistics of NV in healing skin. (*n* = 3 independent samples. *p＜0.05 and **p＜0.01. All statistical analyses were performed by one-way ANOVA. Data are presented as mean ± SEM).  **g** Quantitative statistics of new HF in healing skin. (*n* = 3 independent samples. **p < 0.01. All statistical analyses were performed by one-way ANOVA. Data are presented as mean ± SEM). **h**, **i** Representative fluorescence images and fluorescence intensities of EGFR (red) in wound areatreated with CD, CMNP (EGF), ES, and mn-STESS (EGF and ES). (*n* = 3 independent samples. **p < 0.01 and ***p < 0.001. All statistical analyses were performed by one-way ANOVA. Data are presented as mean ± SEM). Source data are provided as a Source Data file.

Translational medicine results showed mn-STESS significantly promoted wound healing. As shown in Fig. 5a, two CMNPs were attached to both sides of the wound to avoid secondary traumatization in the wound area. mn-STESS continuously released EGF into the skin and provided self-powered ES as an adjuvant for synergistic therapy. The surgical photos showed that the wound healing effect in all intervention groups was superior to that in the CD group. Especially in the mn-STESS group, the healing rate in the first 6 days was ~18% and ~36% faster than that in the CMNP and ES groups, respectively (Fig. 5b–d). H&E staining of tissue from the wound center showed that the newborn epithelium (NE) in the mn-STESS group was longest (Supplementary Fig. 21). H&E staining of wound healing tissue also showed that the mn-STESS group presented even better wound healing quality, with more new vessels (NV) and hair follicles (HF) compared to the other groups (Fig. 5e–g). The above results demonstrated that the electrical adjuvant produced by sf-TENG synergistically enhanced the wound-healing effect of EGF.

To confirm in animal models that the wound-healing effect of mn-STESS was mediated by improvement of EGF pharmacodynamics, we further evaluated EGFR and PI3K expression in wound tissue by immunofluorescence staining. The fluorescence results showed that the expression of EGFR and PI3K in the mn-STESS group were increased by 43% and 55% compared with those in the CMNP group, respectively (Fig. 5h, i and Supplementary Fig. 22). Notably, no obvious receptor desensitization occurred in the new epithelial tissue of the CMNP group, which was attributed to the metabolism and diffusion of EGF in vivo 24 h after administration. However, at the CMNP puncture site, significant EGFR desensitization could still be observed due to the high local drug residual concentration, and the expression of EGFR in the CMNP group was only about 40% of that in the CD group, which was similar to the results of cell experiments. In contrast, the mn-STESS group could still effectively compensate for the desensitization of EGFR at the microneedle puncture site (Supplementary Fig. 23a, b). The consistency of the in vitro and in vivo findings consolidated the compensatory effect of mn-STESS on receptor desensitization and further activated the EGF/EGFR pathway and downstream molecule by improving EGF pharmacodynamics, thereby significantly promoting wound healing.

**Fig. 6:**
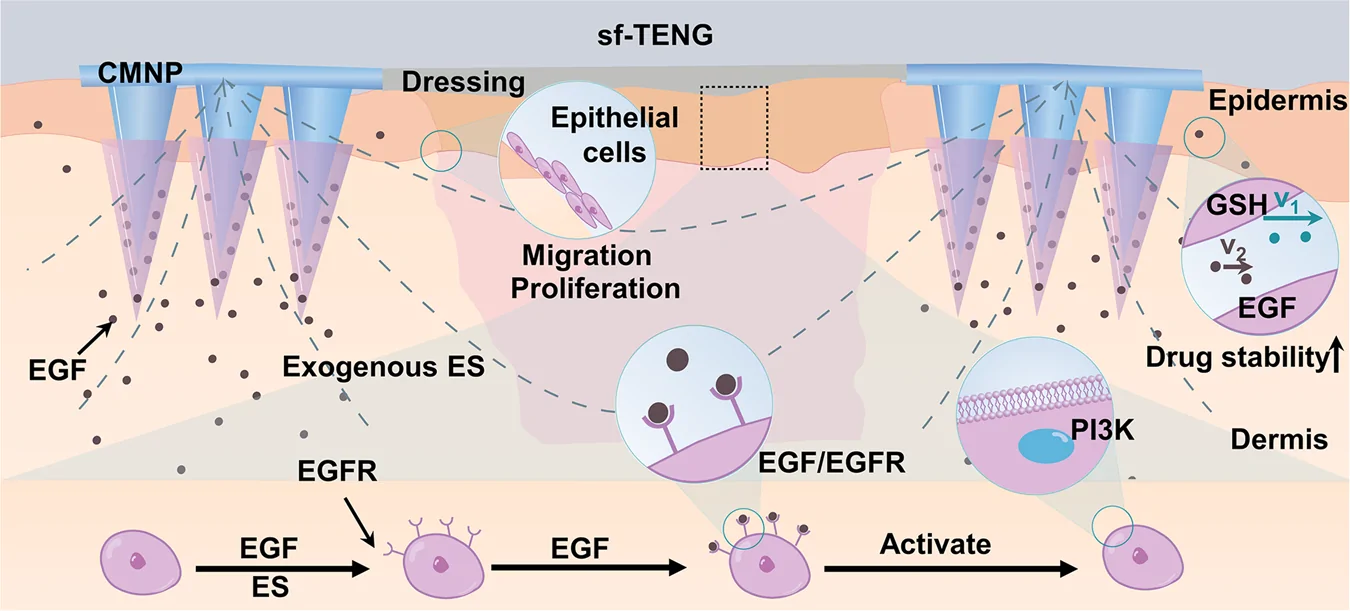
mn-STESS improved EGF pharmacodynamics to promote wound healing. mn-STESS not only delivered EGF transdermally, but also performed transdermal ES, which acted as an adjuvant and synergized with EGF. ES generated by mn-STESS reduced GSH-mediated reduction of EGF to maintain its stability, upregulated cellular EGFR expression to compensate for receptor desensitization, and activated the downstream factor PI3K to promote wound healing.

In summary, we developed the mn-STESS by integrating sf-TENG and CMNP (Fig. 6). The sf-TENG converted the mechanical energy generated by finger sliding into electricity. The two-stage structure of CMNP could not only deliver drugs, but also introduce a microcurrent produced by sf-TENG into the dermis for transcutaneous ES. In response to pharmacodynamic challenges, mn-STESS improved the efficacy of EGF in various aspects: (i) continuously delivered EGF in a minimally invasive manner to improve drug permeability and utilization; (ii) ES generated by mn-STESS increased the intermolecular distance between EGF and GSH by promoting GSH movement, thereby suppressing the reduction of EGF; (iii) self-powered ES also upregulated EGFR expression to compensate for receptor desensitization and improved the efficacy of EGF. In vitro and in vivo experiments confirmed that the strengthened EGF pharmacodynamics by mn-STESS promoted cell proliferation and migration by activating the EGF/EGFR pathway and downstream molecule PI3K. Furthermore, mn-STESS therapy possessed great benefits in wound healing by promoting wound re-epithelialization, vascularization, and HF formation. This work proposed a therapeutic strategy based on self-powered electrical adjuvants, opening a new era for improving the tolerance of classic drugs.

## Methods

### Materials

Polydimethylsiloxane (PDMS, Sylgard 184) was obtained from Dow Corning (Midland, USA). Polylactic acid (PLA) was purchased from Lakeshore Biomaterials Inc. (AL, USA). NaOH was purchased from Shanghai Aladdin Bio-Chem Technology Co., LTD (Shanghai, China). 1,4-butanediol diglycidyl ether (BDDE, Mw = 202.25 Da) was obtained from Adamas Reagent Co. Ltd. (Basel, Switzerland). Genipin was purchased from J&K Scientific (Beijing, China). Gelatin (Gel) from cold water fish skin and from porcine skin, hyaluronic acid (HA, Mw ≈ 20,000–400,000 Da) were purchased from Sigma-Aldrich (MO, USA). Human epidermal growth factor (EGF), FITC-labeled EGF, glutathione, DMEM high glucose medium, penicillin/streptomycin, CCK-8, Triton X-100, DAPI and BSA were purchased from Beijing Solarbio Technology Co., Ltd (Beijing, China). 1640 medium and fetal bovine serum (FBS) were obtained from Gibco. The antibodies used in this study were summarized as follows (category number, company): Phalloidin-iFluor 488 (ab176753, Abcam), anti-EGFR (ab52894, Abcam), anti-PI3 Kinase p110 beta (ab151549, Abcam), Goat Anti-Rabbit IgG H&L (Alexa Fluor 647) (ab150083, Abcam) and Goat Anti-Rabbit IgG H&L (FITC) (ab6717, Abcam).

### Fabrication of MN matrix materials and drug formulations

The cross-linked gelatin (cGel) solution and the cross-linked hyaluronic acid (cHA) microparticle were prepared for encapsulating drug through the chemical cross-linking according to the previous reported method^46^. Briefly, 40% (w/v) Gel solution was obtained by dissolving the Gel powders from cold water fish skin in deionized water under magnetic stirring at 50 °C for 1 h. Then, the genipin solution was slowly dropped dropwise to the prepared Gel solution, and mixed at 40 °C for 96 h to produce the cross-linked Gel. The cHA hydrogel was prepared by mixing hyaluronic acid powder (HA, Mw ≈ 20,000–400,000 Da, 1 g) and 1,4-butanediol diglycidyl ether (BDDE, 200 μl) in NaOH solution (0.25 M, 9.8 mL, pH = 13) at 65 °C for 3 h, and then purifying with 95% alcohol to remove extra NaOH, BDDE and uncrosslinked HA fragments. Finally, the acquired cHA hydrogel was masked by a ball mill for 20 min, and filtered with a 500-mesh filter to obtain cHA gel microparticles. In order to encapsulate the drug, the obtained cHA microparticles were first immersed in human epidermal growth factor (EGF) solution (10 μg mL^−1^, 1 mL) to fully swell to receive drug-loaded microparticles. Then the drug-loaded cHA microparticle and EGF drug power were mixed uniformly with cGel under magnetic stirring at room temperature to obtain the cGel-cHA solution.

### Fabrication of the CMNP

Polylactic acid microneedle patch (PLA MNP) was prepared by thermoforming with the polydimethylsiloxane (PDMS, Sylgard 184) template. PLA-Au MNP were subsequently obtained by sputtering a layer of gold with the thickness of 50 nm on the surface of PLA MNP. The prepared drug solution (10 μg mL^−1^, 100 μL) was applied on the PDMS template under vacuum at −85 kPa for 30 min. Then, the remaining drug was removed from the surface of template, and the drug solution filled in the cavity was dried at room temperature under vacuum at −85 kPa for 20 min. Next, the prepared matrix material (Gel, cGel, and cGel-cHA) was filled into the cavity under vacuum for 45 min. Following removal of residual material from the mold surface, PLA-Au MNP was aligned and pressed into the drug-loaded MN cavity, and dried at room temperature for 12 h. The two stage PLA-Au/cGel-cHA CMNP was successfully removed from the mode for further analysis.

### Characterization of MNP

The size and morphology of MN was observed using a stereomicroscope (SZX7, Olympus, Japan) and a cold field emission scanning electron microscope (SEM, SU8020). The mechanical property of MN was measured using a dynamometer (Force Gauge Model, Mark-10, USA). The MNP was placed on the rigid stainless-steel stage, and the mechanical sensor probe was slowly moved vertically downward at a speed of 1 mm min^−1^ until the set value of 20 N was reached. In this process, the function of force and displacement was recorded. The Young’s modulus of the MN was calculated according to the following formula,

1 $${{{\rm{E}}}}={{{\rm{\sigma }}}}/{{{\rm{\varepsilon }}}}=({{{\rm{F}}}}/{{{\rm{A}}}})/(\Delta {{{\rm{L}}}}/{L}_{0})={{{\rm{F\cdot }}}}{L}_{0}/{{{\rm{A\cdot }}}}\Delta {{{\rm{L}}}}$$

where E is the Young’s modulus, σ is the uniaxial stress, ε is the strain, F is the compressive force, A is the cross-section area perpendicular to the applied force, ΔL is the change in length (negative value if compressed), and L_0_ is the original length.

For the penetration ability of the MNs, the MNP was inserted into penetration artificial membrane using 10 N produced by the mechanics test bench. The artificial membrane with the thickness of 127 μm was made by folding parafilm into an 8-layer square. The MNP was attached to the mechanical sensing probe and vertically pierced into the artificial membrane. After peeling off the MNP, the penetration rate of MNP was determined by accumulating the number of holes on each layer of parafilm, and insertion depth was calculated by adding the depression depth of the last parafilm layer to the thickness of all penetrated parafilm (Supplementary Fig. 24).

2 $${{{\rm{Insertion\; depth}}}}=127\times {{{\rm{piercing\; layers}}}}+{{{\rm{depression\; depth}}}}$$

### Preparation of sf-TENG

sf-TENG consisted of two triboelectric layers and two electrodes. A PI film (30 × 60 mm) and two PVDF films (7 × 7 mm) were utilized as the triboelectric layer and dielectric layers, respectively. Two conducting PLA-Au MNP of CMNP were especially employed as electrodes. The surface charge density of the PTFE dielectric layer was increased by applying the corona discharge method. Briefly, PTFE film was covered on the Al sheet grounded by a wire, and a polarization voltage of 5 kV was loaded on the PTFE film for 15 min through a corona needle. Furthermore, in order to avoid the loss of charge, a layer of PI was covered on PTFE film.

### Characterization of sf-TENG

The output performance of sf-TENG including voltage, current, and transferred charge were measured and recorded by oscilloscope (HD 4096, Teledyne LeCroy). In the test, a finger or the pigskin instead of finger was used to touch sf-TENG driven by a linear motor (E1100, LinMot) with the frequency of 1, 1.5, and 2 Hz.

### Drug delivery in vitro

To determine the release kinetics of CMNP loading EGF, loaded-drug part of CMNP fabricated by the different matrix materials (Gel, cGel, cGel-cHA) were completely immersed in 0.01 M phosphate Buffer solution (PBS) with the magnetically stirring of the 200 rpm at 37 °C for 24 h. At the specific time points, ~300 μL of the sample was extracted and supplemented with the equal volume of fresh PBS. Following the obtained sample filtered with the filter tube (M_W _= 10,000), the release amount of EGF form CMNP was quantitatively tested by the UV absorbance at 278 applying the ultraviolet spectrophotometer (UV/VIS Spectrometer, Lambda 35, PerkinElmer). The drug loading of CMNP was 0.5 μg. The EGF release efficiency of CMNP was determined by the ratio of the cumulative release amount at the different times and the drug loading of CMNP. For the release drug of mn-STESS, two CMNP immersed in PBS were connected to the two electrodes of sf-TENG (the other two CMNPs) through copper wires, and a piece of pig skin (10 × 10 mm) instead of human finger was used to slide on the sf-TENG driven by a linear motor. The activity of EGF released from CMNP and mn-STESS in GSH solution (10 μmol L^−1^, 3 mL) was tested by human EGF ELISA kit.

### Animal experiments

All animal experiments were performed according to protocols approved by approved by Committee on Ethics of Beijing Institute of Nanoenergy and Nanosystems (A-2019027). Mice were maintained on a 12 h light/dark cycle in individually ventilated cages at 22 °C and 48% humidity with unrestricted access to food and water unless otherwise state. Mice were group-housed if same sex little-mates were available. Mice were purchased from the Beijing Vital River Laboratory Animal Technology Co., Ltd., China.

### Drug delivery in vivo

To visualize drug release in vivo, FITC-labeled EGF was loaded into CMNP. Two CMNPs were simultaneously pierced into the dorsal skin without the hair of the female Kunming mice (6–8 weeks, 25–30 g) at a distance of 1 cm. Six mice were treated with mn-STESS for 24 h. The two CMNP applied on skin were connected to the two electrodes of sf-TENG by copper wires. sf-TENG outputted the 1 μA current by using pigskin sliding on it. Fluorescent images of mice were captured using in vivo imaging system (IVIS, Xenogen 200, Caliper Life Sciences, Hopkinton, MA), and the fluorescent intensity of FITC labeled EGF on the insertion area at different times (1, 2, 6, 12, 18, 24 h) were measured using Living Image 4.0 software package. When the mn-STESS was peeled off the skin, the first-stage PLA-Au microneedles were removed along with the patch, and the optical micrographs confirmed that the PLA-Au microneedles were not bent or broken. The second-stage cGel-cHA microneedles were retained in the body and gradually degraded. In vitro experiments confirmed that the cGel-cHA microneedles could be completely degraded after 72 h in PBS. (Supplementary Fig. 25a, b).

### Cell culture

The HaCaT cells (keratinocyte cells, SCSP-5091) and L929 cells (fibroblasts, GNM28) were acquired from the Cell Bank of the Chinese Academy of Sciences in Beijing, China. HaCaT cells were cultured in 1640 medium (C11875500BT) containing 10% fetal bovine serum (FBS) and 1% penicillin/streptomycin (P1400). L929 cells were cultured in DMEM high glucose medium (11995) containing 10% FBS and 1% penicillin/streptomycin (P1400). The cell culture condition was humid incubator (CCL-170B-8, ESCO) with 5% CO_2_ at 37 °C.

### Safety assessment

All materials of mn-STESS were tested for cytotoxicity. The cell viability/cytotoxicity detection and CCK-8 was used to assess L929 cell viability. The images were captured by a confocal fluorescence microscope (Leica SP8). The microplate reader (BioRad iMark) was applied to test absorbance.

### Cell proliferation experiment

The HaCaT cells were cultured in 12-well cell plate with CMNP for 24 h. The 12-well cells were randomly assigned to four groups, namely Blank group, CMNP group, ES group, and mn-STESS group. The cells in Blank group received no intervention. The cells in the CMNP group were treated with 10 ng mL^−1^ EGF. The cells were treated with the sf-TENG at current of 1 μA in the ES group. In the mn-STESS group, cells were treated with mn-STESS which loaded 10 ng mL^−1^ EGF and outputted current of 1 μA. CCK-8 was used to assess the viability of HaCaT cells. The microplate reader (BioRad iMark) was applied to test absorbance.

### Scratch test

The HaCaT cells were cultured in 12-well cell plate with CMNP for 48 h. Whereafter, a scratch about 400 μm wide was made on the HaCaT cells that covered the 12-well cell plate using the tip of the pipette. The cells are intervened under different conditions. HaCaT cells were treated by the 1 μA ES generated by sf-TENG and the 10 ng mL^−1^ EGF from CMNP.

### Immunofluorescence of HaCaT cells

The HaCaT cells were treated by EGF, ES, and mn-STESS in 12-well cell plate, and the Blank group had no intervention. The cells were blocked with 3% BSA (SW3015) and 10% FBS (10099–141, Gibco) in 0.3% Triton X-100 (T8200) for 2 h at room temperature, incubated with Phalloidin-iFluor 488 (ab176753, Abcam, 1:1000), anti-EGFR (ab52894, Abcam,1:200) and anti-PI3 Kinase p110 beta (ab151549, Abcam,1:200) overnight, and washed 3 times with PBS. Then the sections incubated with Goat Anti-Rabbit IgG H&L (Alexa Fluor 647) (ab150083, Abcam, 1:400) and Goat Anti-Rabbit IgG H&L (FITC) (ab6717, Abcam, 1:400) for 1 h. Finally, DAPI (1:100, c0060) was used to incubate the sections. The photos were taken with the confocal fluorescence microscope (Leica SP8). Image–Pro Plus 6.0 was used to analyze the positive expressions.

### Animal experiment for wound healing

The experiments were performed on 36 female Kunming mice (6–8 weeks, 25–30 g) without any skin diseases. The back hair of mice was removed with an electrical hair cutter and depilation cream. The full-thickness skin wound (7 × 7 mm) was excised on the back. The 36 mice were randomly divided into five groups and treated for 12 days (replaced once a day), namely CD group, CD-EGF group, CMNP group, CMNP-sliding group, ES group, and mn-STESS group. The wounds in the CD group were covered with only chitosan dressings. The wounds in CD-EGF group were covered with chitosan dressings loading 1 μg EGF. The wounds in the CMNP group were treated by two CMNPs loading 0.5 μg EGF without sliding. The wounds in the CMNP-sliding group were treated by two CMNPs loading 0.5 μg EGF with sliding. The wounds were treated with the sf-TENG at current of 1 μA in the ES group. In the mn-STESS group, wounds were treated with mn-STESS which loaded 1 μg EGF and outputted current of 1 μA. On the 0th, 3rd, 6th, 9th and 12th day, the wound form was observed and recorded by a digital camera. Image–Pro Plus 6.0 was used to measure surface areas of the wounds. On the 12th day (without treatment), wound tissues were collected for observation and analysis.

3 $${{{\rm{Remaining}}}}\;{{{\rm{wound}}}}\;{{{\rm{area}}}}\,(\%)=[{{{\rm{wound}}}}\;{{{\rm{area}}}}\big/{{{\rm{initial}}}}\;{{{\rm{wound}}}}\;{{{\rm{area}}}}]\times 100$$

### Wound electrical measurement

The wound potential and current were measured by the electrometer (Keithley 6517B) and oscilloscope (Teledyne LeCroy HD 4096). The positive electrode of the potential electrode needed to be placed on the wound edge and the negative electrode needed to be placed on the wound center.

### Histology

Four percent paraformaldehyde was used to soak tissues overnight. The tissues were dehydrated with graded ethanol and embedded in paraffin blocks for sectioning at 4 μm. Tissue sections were stained with hematoxylin and eosin (H&E) and immunofluorescence (IF). In the IF staining, the sections were blocked with 3% BSA (SW3015) and 10% FBS (10099-141, Gibco) in 0.3% Triton X-100 (T8200) for 2 h at room temperature, incubated with anti-EGFR (ab52894, Abcam, 1:100) and anti-PI3 Kinase p110 beta (ab151549, Abcam, 1:100) overnight, washing 3 times with PBS. Then the sections incubated with Goat Anti-Rabbit IgG H&L (Alexa Fluor 647) (ab150091, Abcam, 1:100) for 1 h. Finally, DAPI (1:100, c0060) was used to incubate the sections. The photos were taken with the confocal fluorescence microscope (Leica SP8). Image–Pro Plus 6.0 was used to analyze the positive expressions.

### Statistics and reproducibility

At least three independent experiments of each type have been done and produced consistent results. Specifically, the experiments shown in the following figures were repeated three times: main Figs. 1c–e; 3e, supplementary Figs. 2; 4c; 6b; 7b, c, f; 24b, c; 25a. Statistics are expressed as the mean ± standard error of mean (SEM) of at least three or more independent simple. The one-way ANOVA was used to determine the statistical significance of the differences. Image–Pro Plus 6.0, Origin 2018 and Excel was used for data analysis and plotting. *p < 0.05, **p < 0.01 and ***p < 0.001 were considered statistically significant.

### Reporting summary

Further information on research design is available in the Nature Portfolio Reporting Summary linked to this article.

## Supplementary information

Supplementary Information

Description of Additional Supplementary Files

Supplementary Movie S1

Reporting Summary

## Source data

Source Data

## Data Availability

The authors declare that all data supporting the results in this study are available within the paper and its Supplementary Information, or from the corresponding authors upon reasonable request. Source data for the figures in this study are available from figshare with the identifier https://doi.org/10.6084/m9.figshare.21411567. Source data are provided with this paper.
