# Author Correction: Improved pharmacodynamics of epidermal growth factor via microneedles-based self-powered transcutaneous electrical stimulation

**DOI:** 10.1038/s41467-026-78566-x

**Published:** 2026-10-13

**Authors:** Yuan Yang, Ruizeng Luo, Shengyu Chao, Jiangtao Xue, Dongjie Jiang, Yun Hao Feng, Xin Dong Guo, Dan Luo, Jiaping Zhang, Zhou Li, Zhong Lin Wang

**Affiliations:** 1 Beijing Institute of Nanoenergy and Nanosystems, Chinese Academy of Sciences, Beijing, 101400 China; 2 Department of Plastic Surgery, State Key Laboratory of Trauma, Burns and Combined Injury, Southwest Hospital, Third Military Medical University (Army Medical University), Chongqing, 400038 China; 3 School of Nanoscience and Technology, University of Chinese Academy of Sciences, Beijing, 100049 China; 4 Institute of Engineering Medicine, Beijing Institute of Technology, Beijing, 100081 China; 5 Beijing Laboratory of Biomedical Materials, College of Materials Science and Engineering, Beijing University of Chemical Technology, Beijing, 100029 China; 6 Center of Nanoenergy Research, School of Physical Science and Technology, Guangxi University, Nanning, 530004 China; 7 Institute for Stem Cell and Regeneration, Chinese Academy of Sciences, Beijing, 100101 China; 8 Georgia Institute of Technology, Atlanta, GA 30332 0245 USA

**Keywords:** Biomedical engineering, Bionanoelectronics, Drug delivery, Protein delivery

Correction to: *Nature Communications* https://doi.org/10.1038/s41467-022-34716-5, Article published online 14 November 2022

In the version of the article initially published, there were errors due to mistakes during figure preparation and data processing. In the bright-field and fluorescence channel overlay plot shown in Fig. 3e, the bright-field background images at 1 hour and 24 hours were inadvertently duplicated from the 6-hour background image. In Supplementary Fig. 13b, a column of data was incorrectly pasted repeatedly, resulting in a reported sample size of *n* = 4 instead of *n* = 3 as indicated in the manuscript. In Fig. 3b–3d and Supplementary Fig. 10d, improper Excel formatting caused the last two significant digits in the original data to be missing. In the Source Data for Figs. 4c, 4d, 5f, 5i and Supplementary Figs. 9a, 10a–c, 10e, unnecessary significant digits (i.e., unnecessary zeros) were automatically added to the original data due to Excel formatting settings. The label “F-actin” in Fig. 4b was incorrectly spelled as “F-action”. The figures and Source Data have been corrected in the HTML and PDF versions of the article. These changes do not affect the results or conclusions. The original figures are available as Supplementary Information accompanying this amendment.

## Supplementary information

Original Figs. 3, 4, Supplementary Figs. 10, 13

