# Supplementary material for: Improved pharmacodynamics of epidermal growth factor via microneedles-based self-powered transcutaneous electrical stimulation
Source: Nat Commun. 2022 Nov 14;13:6908. doi: 10.1038/s41467-022-34716-5 (PMC9663450; doi:10.1038/s41467-022-34716-5)
Supplement: Supplementary file 1 — Supplementary Information [file 41467_2022_34716_MOESM1_ESM.pdf]

Supplementary Information

Improved pharmacodynamics of epidermal growth factor via microneedles-based self-powered transcutaneous electrical stimulation

Yuan Yang[1, 2, 3#], Ruizeng Luo[1, 3#], Shengyu Chao[1, 3], Jiangtao Xue[4], Dongjie Jiang[1, 3], Yun Hao Feng[5], Xin Dong Guo[5], Dan Luo[1 ,3, 6], Jiaping Zhang[2]*, Zhou Li[1, 3, 6, 7]*, Zhong Lin Wang[1, 8]*

[1]Beijing Institute of Nanoenergy and Nanosystems, Chinese Academy of Sciences, Beijing 101400, China.

[2]Department of Plastic Surgery, State Key Laboratory of Trauma, Burns and Combined Injury, Southwest Hospital, Third Military Medical University (Army Medical University), Chongqing, China.

[3]School of Nanoscience and Technology, University of Chinese Academy of Sciences, Beijing 100049, China.

[4]Institute of Engineering Medicine, Beijing Institute of Technology, 100081, China.

[5]Beijing Laboratory of Biomedical Materials, College of Materials Science and Engineering, Beijing University of Chemical Technology, Beijing 100029, China.

[6]Center of Nanoenergy Research, School of Physical Science and Technology, Guangxi University Nanning 530004, China.

[7]Institute for Stem Cell and Regeneration, Chinese Academy of Sciences, Beijing 100101, China.

[8]Georgia Institute of Technology Atlanta, GA 30332 0245, USA.

#These authors contributed equally.

Corresponding authors: Dan Luo (email: luodan@binn.cas.cn), Jiaping Zhang (email: japzhang@aliyun.com), Zhou Li (email: zli@binn.cas.cn)

Supplementary Figure 1 ~ Figure 23

Supplementary Table 1 ~ Table 3

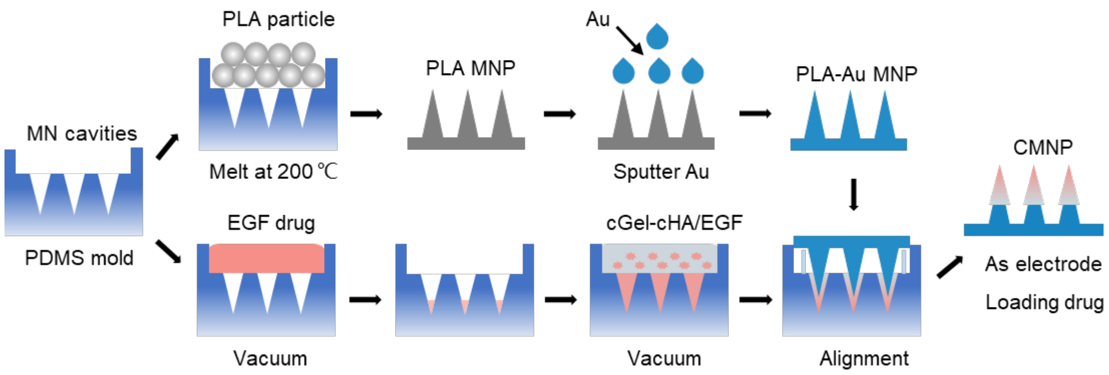

Supplementary Figure 1. Preparation process of CMNP. PLA MNP was prepared by thermoforming at 200°C. PLA-Au MNP were subsequently obtained by sputtering a layer of gold with the thickness of 50 nm on the surface of PLA MNP. The prepared EGF solution (10 μg mL^{-1}, 100 μL) was applied on the PDMS template under vacuum at -85 kPa for 30 min. Then, the remaining drug was removed from the surface of template, and the drug solution filled in the cavity was dried at room temperature under vacuum at -85 kPa for 20 min. Next, the prepared matrix material mixing EGF (cGel-cHA/EGF) was filled into the cavity under vacuum for 45 min. Following removal of residual material from the mold surface, PLA-Au MNP was aligned and pressed into the drug-loaded MN cavity, and dried at room temperature for 12 h. The two stage PLA-Au/cGel-cHA CMNP was successfully removed from the mode for further analysis.

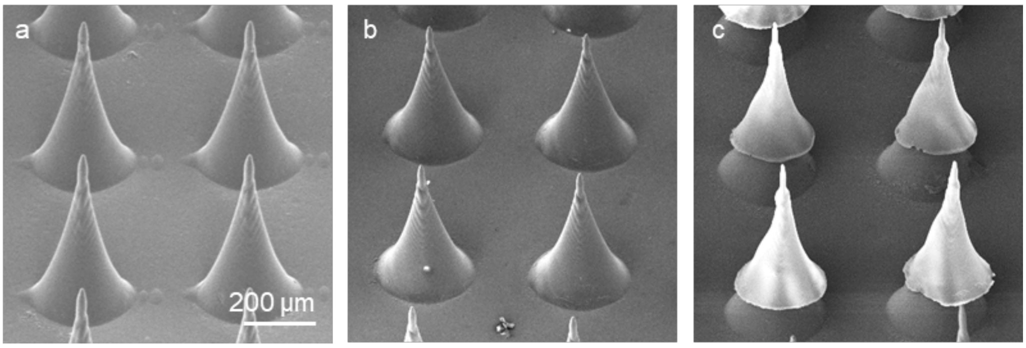

Supplementary Figure 2. The morphology of MNP. (a) SEM images of PLA MNP. (b) SEM images of PLA-Au MNP. (c) SEM images of CMNP.

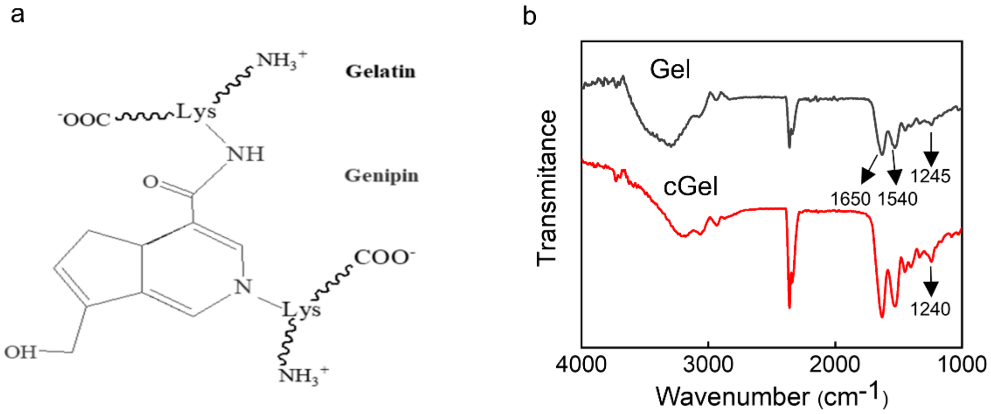

Supplementary Figure 3. Characterization of cGel. (a) Presumable reaction mechanism of genipin with Gel. (b) FT-IR spectra of Gel and cGel. Source data are provided as a Source Data file.

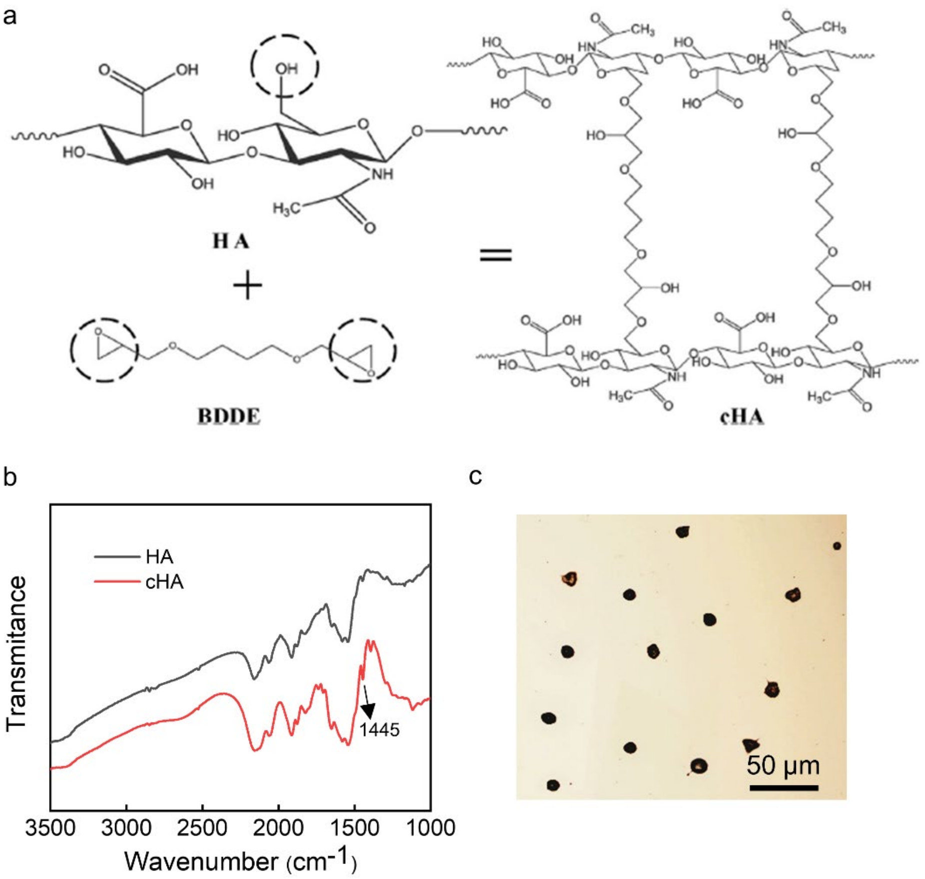

Supplementary Figure 4. Characterization of cHA microparticle. (a) Presumable reaction mechanism of BDDE with HA. (b) FT-IR spectra of HA and cHA. (c) The images of cHA microparticle in bright field. Source data are provided as a Source Data file.

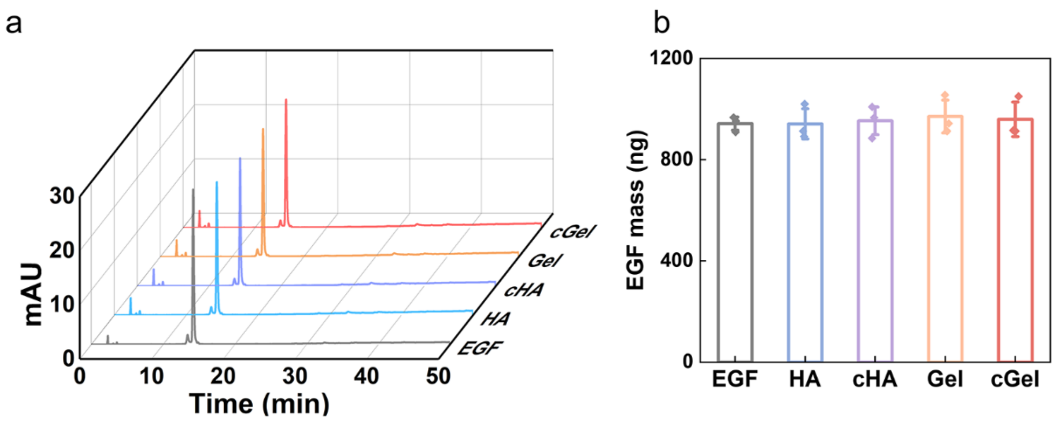

Supplementary Figure 5. EGF activity test before and after cGel and cHA cross-linked**.** (a) high performance liquid chromatography (HPLC) curves of EGF from HA, cHA microparticle, Gel and cGel, EGF solution as blank group. (b) Activity test of EGF from HA, cHA microparticle, Gel and cGel by ELISA, EGF solution as blank group. (n=3 independent samples. Data are presented as mean ± SEM). EGF were measured by high performance liquid chromatography (HPLC) at 278 nm, using a column (Shim-pack GIST C18-AQ (4.6×250 mm, 5μm; P/N: 227-30742-08). Solvent A (0.1% TFA in water) and solvent B (0.1% TFA in acetonitrile) were used as the mobile phase. Source data are provided as a Source Data file.

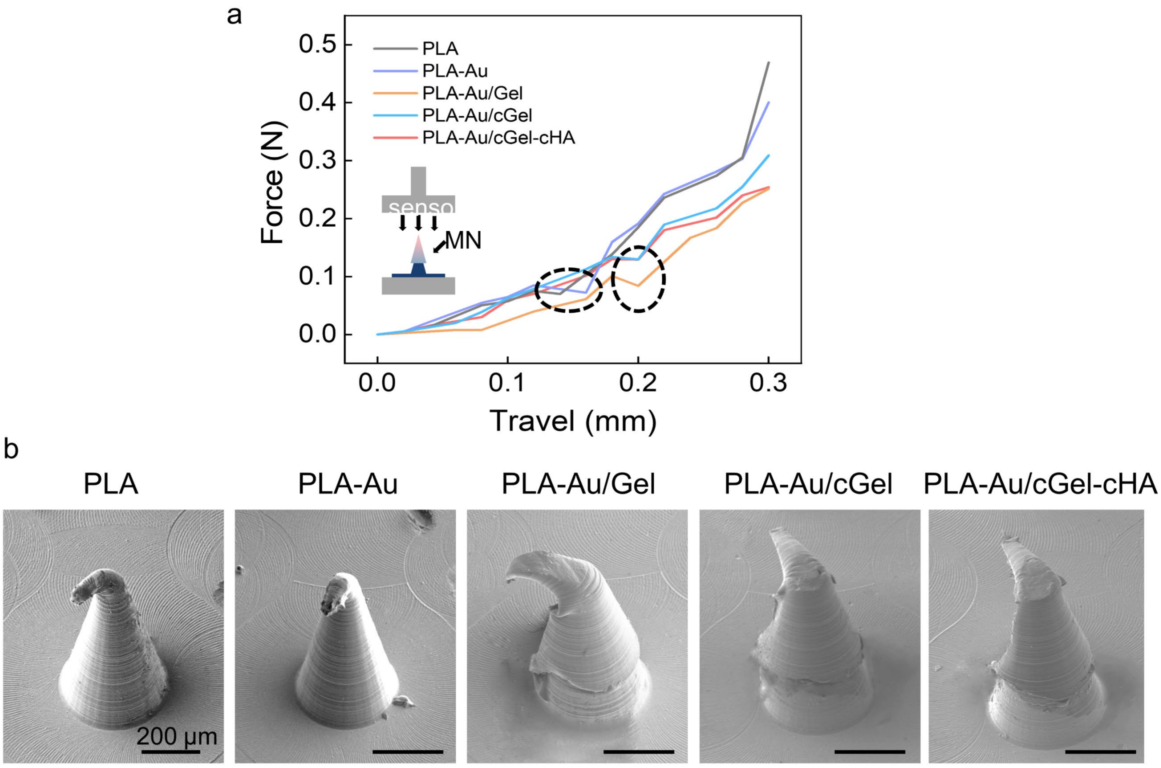

Supplementary Figure 6. Mechanical properties characterization of single microneedle. (a) Typical force-displacement curve of a single microneedle, (inset) schematic of the experimental setup. Black dotted area indicated tip breaking point. (b) SEM images of PLA MN, PLA-Au MN, PLA-Au/Gel MN, PLA-Au/cGel and PLA-Au/cGel-cHA after force measurement. Source data are provided as a Source Data file.

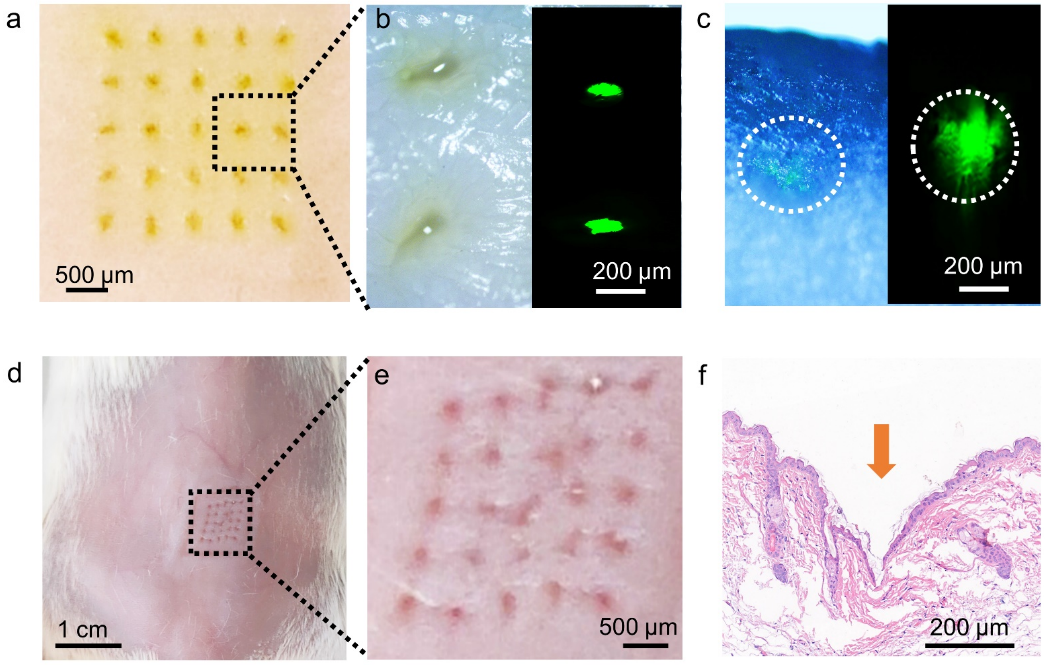

Supplementary Figure 7. Penetration ability of CMNP. (a) Porcine cadaver skin after treatment with CMNP. (b) Magnified images of porcine cadaver skin after treatment with CMNP in brightfield (left) and fluorescence (right) fields. (c) Cross-sectional view of porcine cadaver skin in brightfield (left) and fluorescence (right) field. (d and e) Mouse dorsum after treatment with an CMNP (f) H&E staining of the mouse skin after insertion of microneedles.

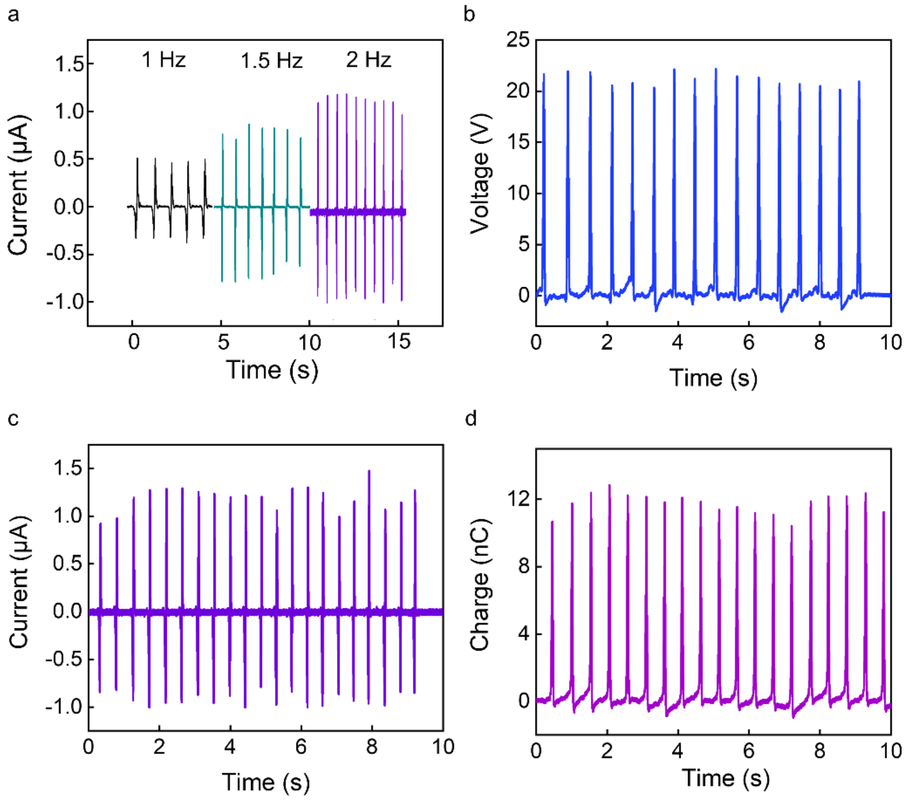

Supplementary Figure 8. sf-TENG output performance. (a) I_{SC} of the sf-TENG at different sliding frequencies. A frequency of 2 Hz was the acceptable optimum value of finger sliding on sf-TENG. (b-d) V_{OC}, I_{SC}, and Q_{SC} of the sf-TENG by the finger sliding. Source data are provided as a Source Data file.

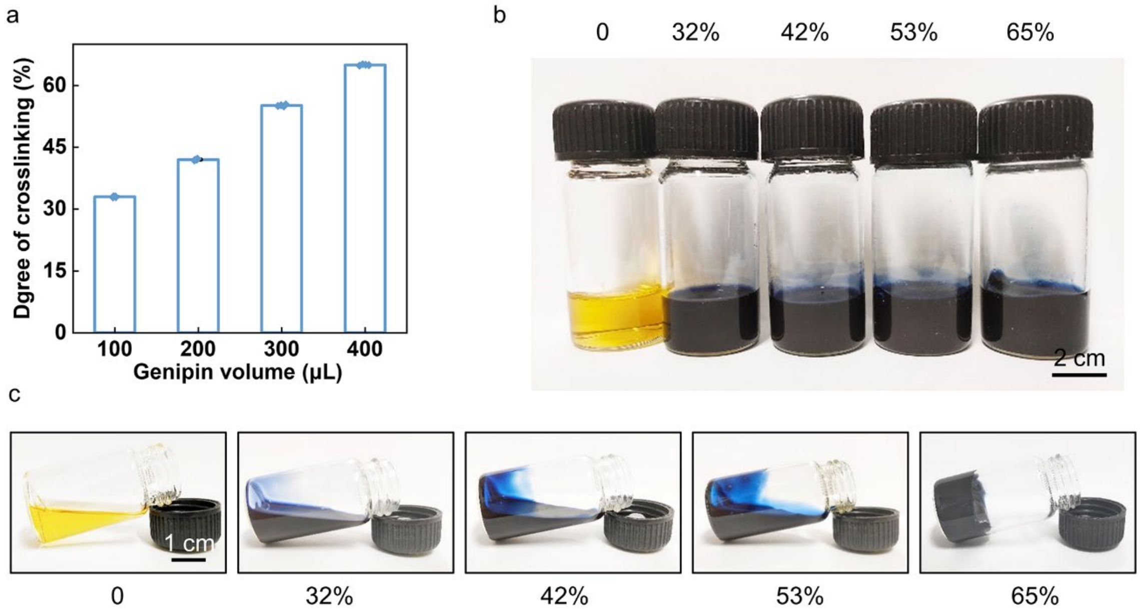

Supplementary Figure 9. cGel with different cross-linked degrees. (a) The cross-linked degree of Gel solution with the different volumes of genipin solutions. (n=4 independent samples. Data are presented as mean ± SEM). (b) Image of Gel solution with the different cross-linked degrees 0, 32%, 42%, 53%, 65%. (c) Fluidity presentation of Gel solutions with the different cross-linked degrees 0, 32%, 42%, 53%, 65%. Source data are provided as a Source Data file.

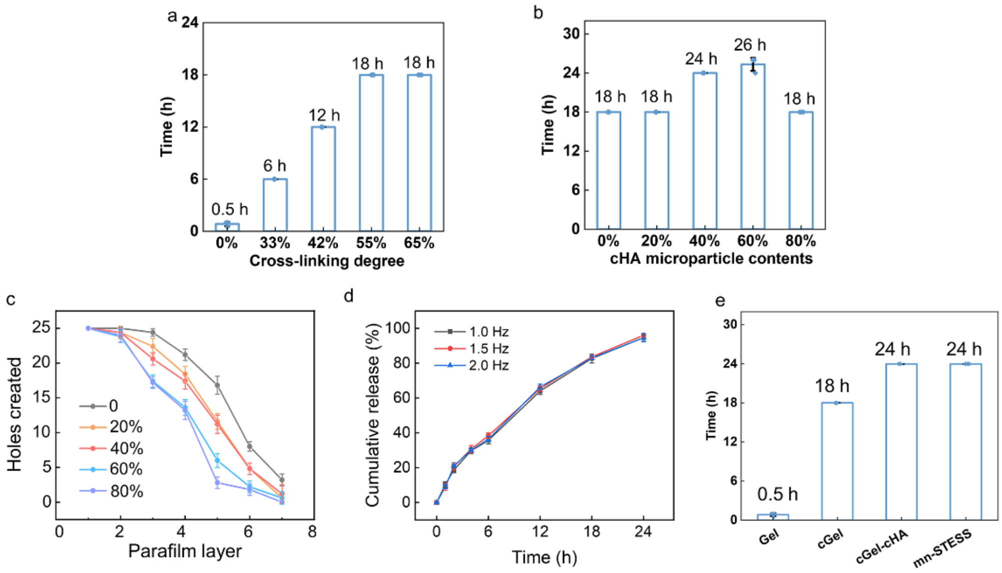

Supplementary Figure 10. EGF release rate. (a) The sustained release time of Gel MN with the different cross-linked degrees 0, 32%, 42%, 53%, 65%. (n=3 independent samples. Data are presented as mean ± SEM). (b)The sustained release time of cGel-cHA MN with the different cHA microparticle contents 0, 20%, 40%, 60%, 80%. (n=3 independent samples. Data are presented as mean ± SEM). (c) Holes created in each parafilm layer after insertion of the cGel-cHA MN with the different cHA microparticle contents. (n=5 independent samples. Data are presented as mean ± SEM). (d) EGF release efficiency from mn-STESS with different sliding frequencies. (n=3 independent samples. Data are presented as mean ± SEM). (e) The sustained release time of EGF from Gel MN, cGel MN, cGel-cHA MN and mn-STESS. (n=3 independent samples. Data are presented as mean ± SEM). Source data are provided as a Source Data file.

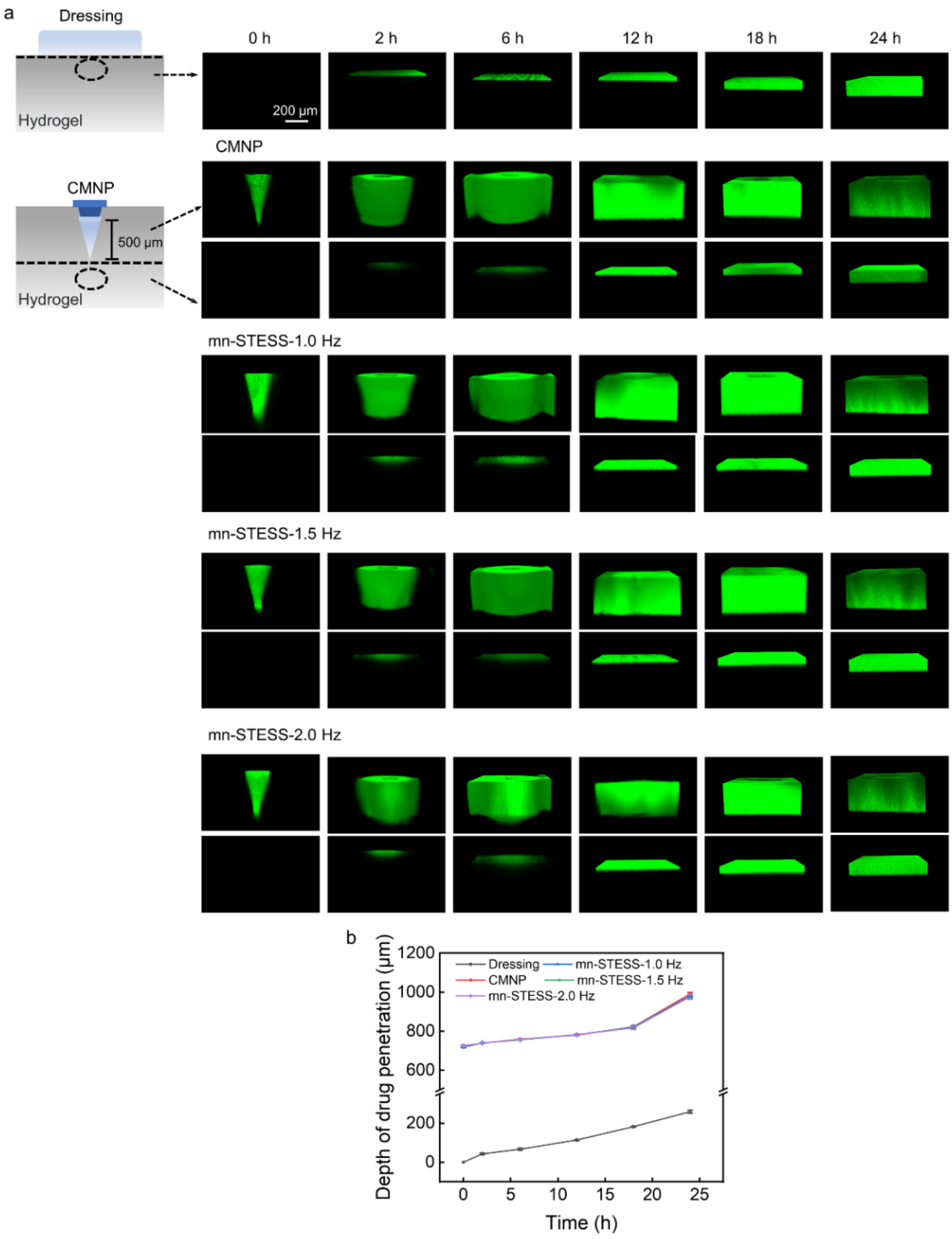

Supplementary Figure 11. The penetration depth and release kinetics of the drug *in vitro*. (a) Fluorescence images of FITC-labeled EGF release from dressing, CMNP and mn-STESS with different sliding frequencies in skin-simulating hydrogels. (b) Penetration depth of FITC-labeled EGF release from dressing, CMNP and mn-TENSS with different sliding frequencies in skin-simulating hydrogels. (n=3 independent samples. Data are presented as mean ± SEM).

A hydrogel prepared from 15% porcine gelatin was used to simulate skin, where a 500 Da dialysis bag was overlaid on the hydrogel to simulate the stratum corneum during dressing

administration. The penetration process of FITC-labeled EGF was observed using a confocal microscope. For CMNP and mn-STESS groups, due to the limitation of the depth of the microscope, taking the needle tip as the boundary, the fluorescence images were taken twice, the upper layer was the lateral diffusion range of the needle body, and the lower layer was the downward diffusion depth of the drug. Source data are provided as a Source Data file.

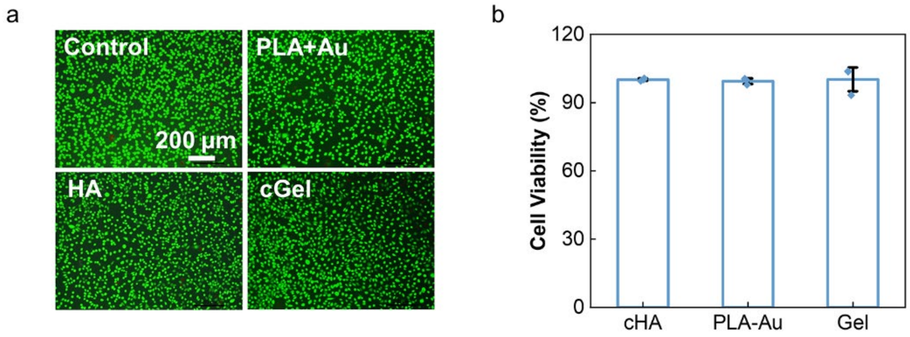

Supplementary Figure 12. Material toxicity test. (a) Fluorescence images of L929 cells after treating by the various matrix material of CMNP for 24 h. (b) Relative viability of L929 cells incubated with the various matrix material of CMNP for 24 h. (n=3 independent samples. Data are presented as mean ± SEM). Source data are provided as a Source Data file.

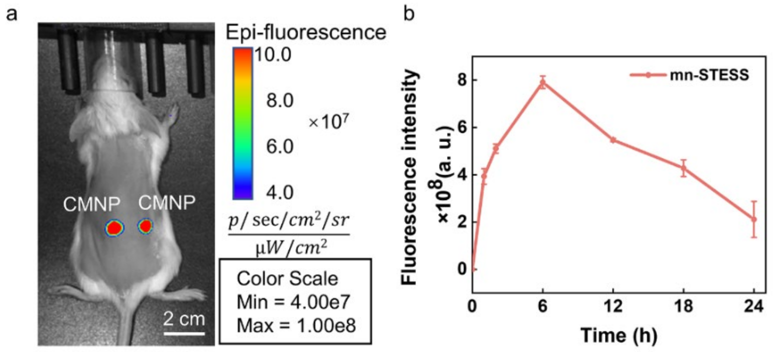

Supplementary Figure 13. Drug delivery *in vivo*. (a) Fluorescence images of skin site penetrated by CMNP. (b) Fluorescence intensity of skin site penetrated by mn-STESS at different times, CMNP as control group. (n=3 independent samples. Data are presented as mean ± SEM). Source data are provided as a Source Data file.

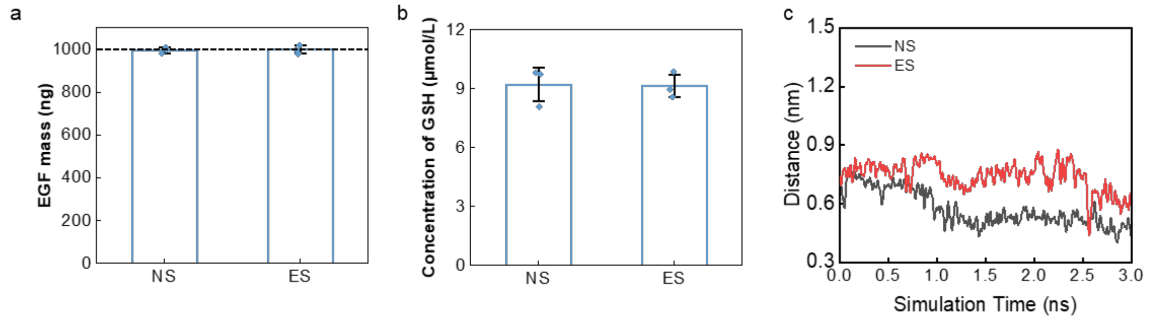

Supplementary Figure 14. Activity testing of EGF. (a) Mass of EGF with ES from sf-TENG, NS (without ES) as control group. (n=3 independent samples. Data are presented as mean ± SEM). (b) Concentration of GSH under ES from sf-TENG, NS as control group. (n=3 independent samples. Data are presented as mean ± SEM). (c) Distance between EGF molecule and GSH molecule under ES from sf-TENG. A simulation box with a side length of 6 nm has been constructed with one EGF molecule and 200 GSH molecules. After solvation, energy minimization and adequate pre-equilibration, the minimum distance between the diffusion of each molecule and the reactive group has been calculated by all-atom NPT molecular dynamics simulation. For the group with current, an electric field strength of 0.0069 V/nm has been assigned in the z-axis direction to simulate the effect of current. Source data are provided as a Source Data file.

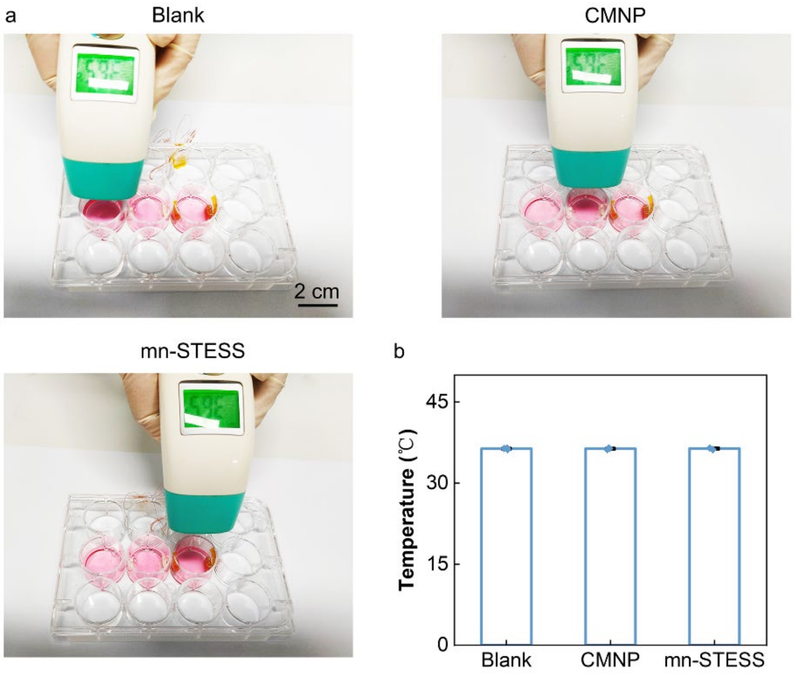

Supplementary Figure 15. Temperature of the culture medium. (a, b) The temperature test pictures and statistics of the cell culture medium in CMNP and mn-STESS groups, untreated culture medium as Blank group. (n=3 independent samples. Data are presented as mean ± SEM). The theoretical heat energy generated by mn-STESS was calculated according to the following formula, $W_h = \eta R \int_0^{t_0} J^2(t)dt$. Where W_h is the thermal energy produced by the current *I* in the time *t,* the resistance of the load is R, and η is the thermal efficiency of the current. Source data are provided as a Source Data file.

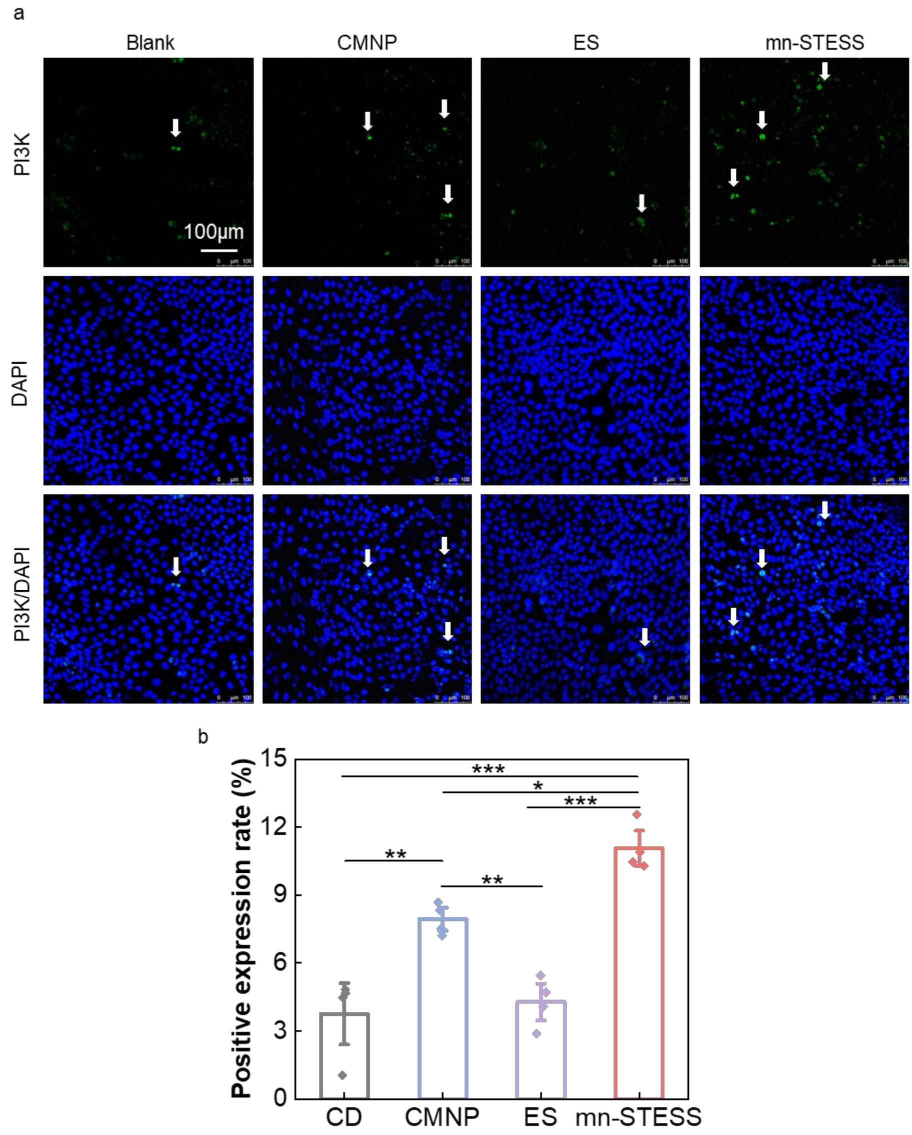

Supplementary Figure 16. Expression of PI3K in HaCaT cell. (a, b) Representative fluorescence images and positive expression rate of PI3K (green) in HaCaT cell after treating by CMNP, ES and mn-STESS. (n=4 independent samples. * $p < 0.05$, ** $p < 0.01$ and *** $p < 0.001$. All statistical analyses were performed by one-way ANOVA. Data are presented as mean ± SEM). Source data are provided as a Source Data file.

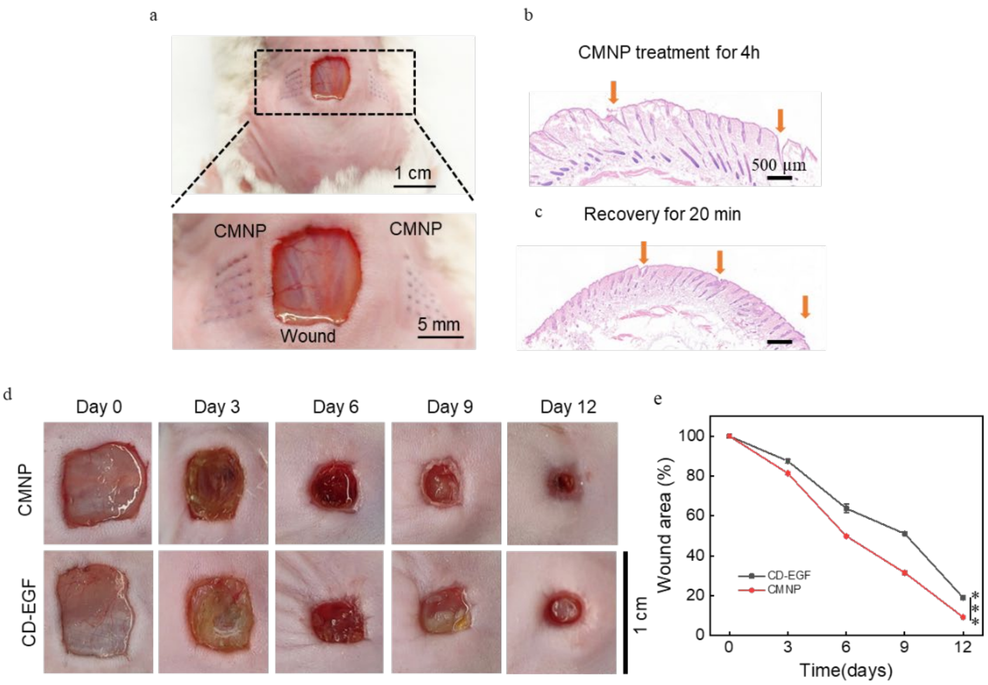

Supplementary Figure 17. Mouse skin after treatment with CMNP. (a) Images of wound model on mouse dorsum and treatment with two CMNPs. (b, c) H&E staining images of mouse skin treated with CMNP for 4 h and recover 20 min after removing CMNP. Scale bar, 500 μm. (d) Representative digital images of the wound area treated by CD-EGF and CMNP on day 0, day 3, day 6, day 9, and day 12. (e) Quantitative statistics of the wound area after treating by CD-EGF and CMNP. (n=4 independent samples. *** $p < 0.001$. All statistical analyses were performed by one-way ANOVA. Data are presented as mean ± SEM). Source data are provided as a Source Data file.

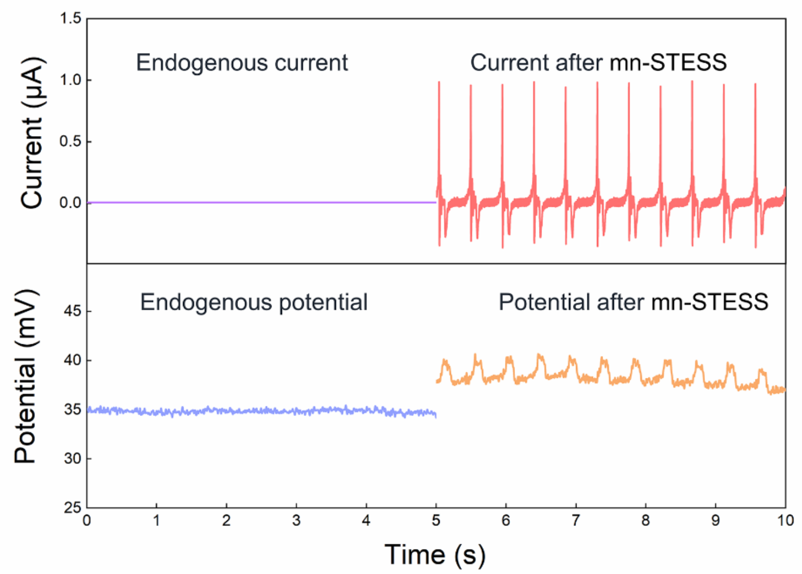

Supplementary Figure 18. Wound currents and potentials before and after mn-STESS intervention. Source data are provided as a Source Data file.

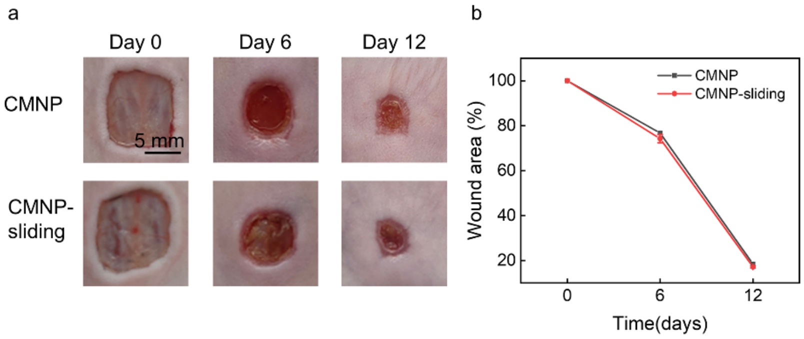

Supplementary Figure 19. Mouse skin after treatment with CMNP. (a) Representative digital images of the wound area treated by CMNP without and with sliding on day 0, day 6 and day 12. (b) Quantitative statistics of the wound area after treating by CMNP without and with sliding. (n=3 independent samples. Data are presented as mean ± SEM). Source data are provided as a Source Data file.

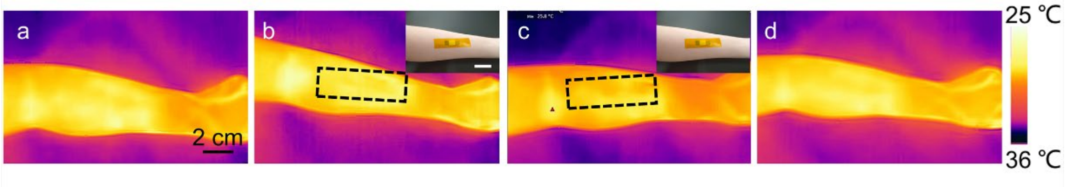

Supplementary Figure 20. Infrared thermal image showing temperature changes, (a) Near-infrared thermal image of normal skin. (b) Near-infrared thermal image of skin with mn-STESS. (inset) Photograph of skin with mn-STESS, scale bar, 2 cm. (c) Near-infrared thermal image of skin with mn-STESS after the finger sliding for 4 h. (inset) Photograph of skin with mn-STESS after the finger sliding for 4 h. (d) Near-infrared thermal image of skin after removal of mn-STESS.

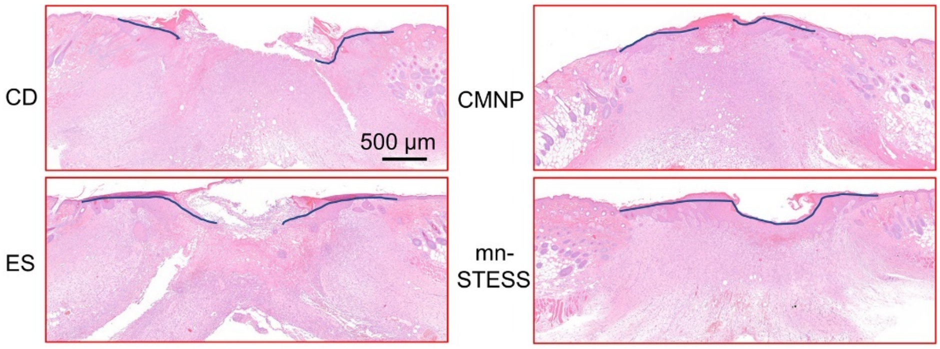

Supplementary Figure 21. H&E staining of the wound center after treating by CMNP, ES and mn-STESS for day 12 (n = 3), CD as control group.

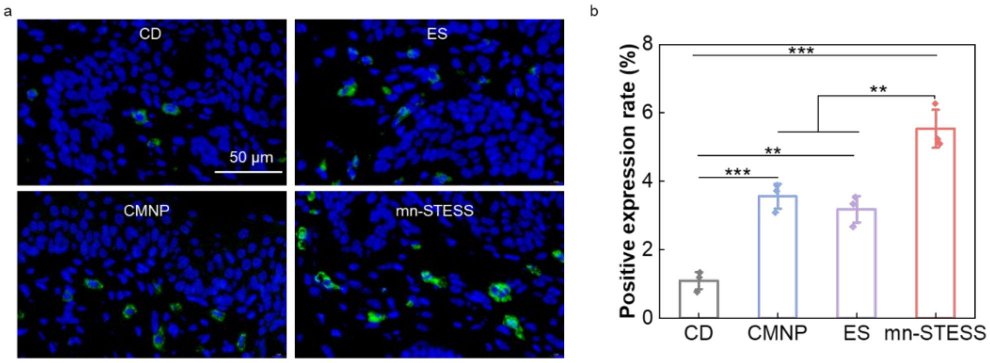

Supplementary Figure 22. Expression of PI3K in healing skin. (a, b) Representative fluorescence images and positive expression rate of PI3K (green) in the wound healing site after treating by CD, CMNP, ES and mn-STESS. (n=3 independent samples. ** $p < 0.01$ and *** $p < 0.001$. All statistical analyses were performed by one-way ANOVA. Data are presented as mean ± SEM). Source data are provided as a Source Data file.

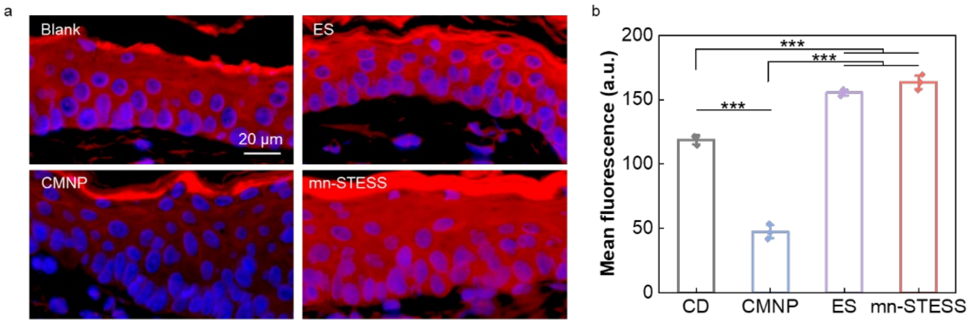

Supplementary Figure 23. EGFR expression at the administration site. (a, b) Representative fluorescence images and fluorescence intensities of EGFR (red) at the site of administration around the wound. (n=3 independent samples. *** $p < 0.001$. All statistical analyses were performed by one-way ANOVA. Data are presented as mean ± SEM). Source data are provided as a Source Data file.

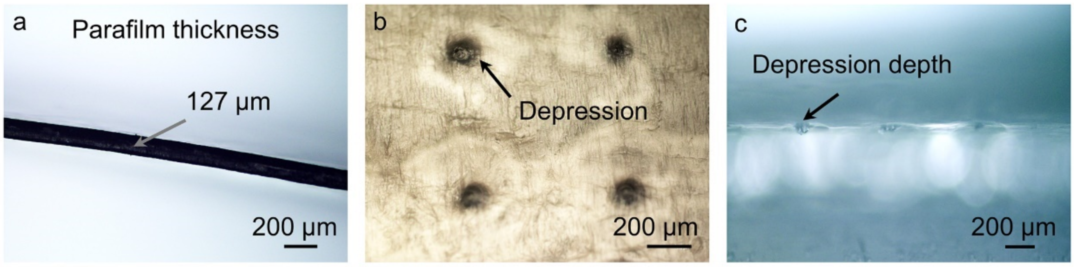

Supplementary Figure 24. Penetration depth of MNs. (a) Optical picture of parafilm. (b, c) Top and cross-sectional views of the last layer of parafilm penetrated by MNs. Insertion depth was calculated by adding the depression depth of the last parafilm layer to the thickness of all penetrated parafilm.

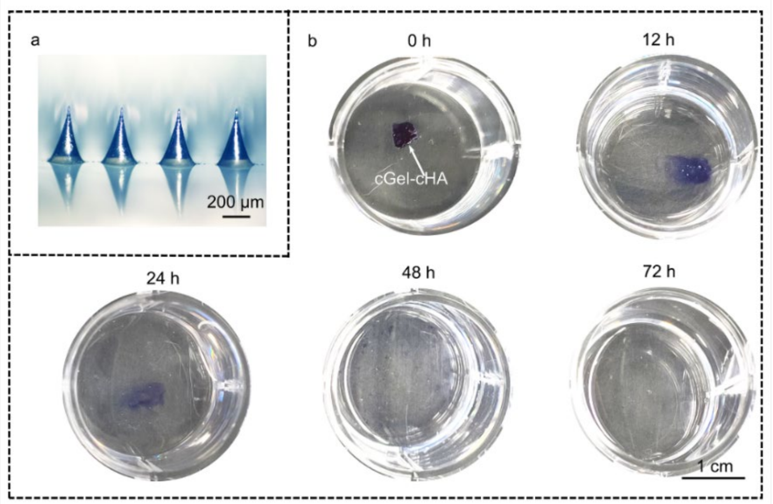

s

Supplementary Figure 25. (a) Brightfield microscopy images of PLA-Au MNP after removing mn-STESS from mouse skin. (b) Degradation process of cGel-cHA film in PBS. The degradation process of cGel-cHA has already been confirmed. cGel is first hydrolyzed into fragments and then further degraded into amino acids, most of which are reused for new protein synthesis, and the remaining amino acids are transaminated to form urea and excreted from the body[1, 2]. cHA could be degraded into glucuronic acid and N-acetylglucosamine in cell lysosomes, and finally decomposed into small molecules by oxidation[3, 4].

Supplementary Table 1 EGF release efficiency from the cGel MNs with the different crosslinking degree.

Time (h)	Cumulative release (%) Mean				
	0	33%	42%	53%	68%
0	0.00	0.00	0.00	0.00	0.00
0.5	88.00	22.13	15.13	9.13	5.27
1	95.67	40.97	30.20	17.47	10.70
1.5	98.33	60.80	40.53	27.20	15.17
2	98.00	75.33	49.00	36.33	22.60
4	98.00	86.00	66.67	51.67	45.00
6	98.27	93.33	85.33	70.67	65.03
12	98.67	96.67	91.67	87.33	85.03
18	98.67	96.33	96.67	98.33	94.60
24	98.97	97.67	98.34	98.00	98.50

Supplementary Table 2 EGF release efficiency from the cGel MNs with the different cHA microparticle contents.

Time (h)	Cumulative release (%) Mean				
	0	20%	40%	60%	80%
0	0.00	0.00	0.00	0.00	0.00
0.5	9.13	5.67	3.67	2.00	6.00
1	17.47	13.67	8.00	5.33	10.67
1.5	27.20	22.337	11.00	7.67	14.67
2	36.33	30.337	20.33	11.33	25.00
4	51.67	40.337	29.00	16.33	33.33
6	70.67	50.00	34.67	23.00	48.00
12	87.33	74.67	64.67	55.33	72.00
18	98.33	93.33	85.00	75.00	92.67
24	98.00	98.00	96.33	86.33	95.67

Supplementary Table 3 EGF release efficiency from Gel MNs, cGel MNs, cGel-cHA MNs, and mn-STESS.

Time (h)	Cumulative release (%) Mean			
	Gel	cGel	cGel-cHA	mn-STESS
0	0.00	0.00	0.00	0.00
0.5	88.00	9.13	3.67	5.33
1	95.67	17.47	8.00	9.00
1.5	98.33	27.20	11.00	13.33
2	98.00	36.33	20.33	21.00
4	98.00	51.67	29.00	30.00
6	98.27	70.67	34.67	36.33
12	98.67	87.33	64.67	66.33
18	98.67	98.33	85.00	83.00
24	98.97	98.00	96.33	94.67

Supplementary References

1. Paguirigan, A. L., Beebe, D. J. Protocol for the fabrication of enzymatically crosslinked gelatin microchannels for microfluidic cell culture. *Nat. protoc.* **2**, 1782-1788 (2007).
2. Jiang, L. B. et al. Salt-assisted toughening of protein hydrogel with controlled degradation for bone regeneration. *Adv. Funct. Mater.* **29**, 1901314 (2019).
3. Khetan, S., et al. Degradation-mediated cellular traction directs stem cell fate in covalently crosslinked three-dimensional hydrogels. *Nat. Mater.* **12**, 458-465 (2013).
4. Al-Sibani, M., Al-Harrasi, A., Neubert, R. H. H. Evaluation of in-vitro degradation rate of hyaluronic acid-based hydrogel cross-linked with 1, 4-butanediol diglycidyl ether (BDDE) using RP-HPLC and UVeVis spectroscopy. *J. Drug Deliv. Sci. Technol,* **29**, 24e30 (2015).
