# Supplementary material for: Improved pharmacodynamics of epidermal growth factor via microneedles-based self-powered transcutaneous electrical stimulation
Source: Nat Commun. 2022 Nov 14;13:6908. doi: 10.1038/s41467-022-34716-5 (PMC9663450; doi:10.1038/s41467-022-34716-5)
Supplement: Supplementary file 2 — Description of Additional Supplementary Files [file 41467_2022_34716_MOESM2_ESM.pdf]

Description of Additional Supplementary Files

Supplementary Movie 1: Output of sf-TENG by finger sliding.
