# Supplementary material for: Author Correction: Improved pharmacodynamics of epidermal growth factor via microneedles-based self-powered transcutaneous electrical stimulation
Source: Nat Commun. 2026 Oct 13;17:10737. doi: 10.1038/s41467-026-78566-x (PMC13662204; doi:10.1038/s41467-026-78566-x)
Supplement: Supplementary file 1 [file 41467_2026_78566_MOESM1_ESM.pdf]

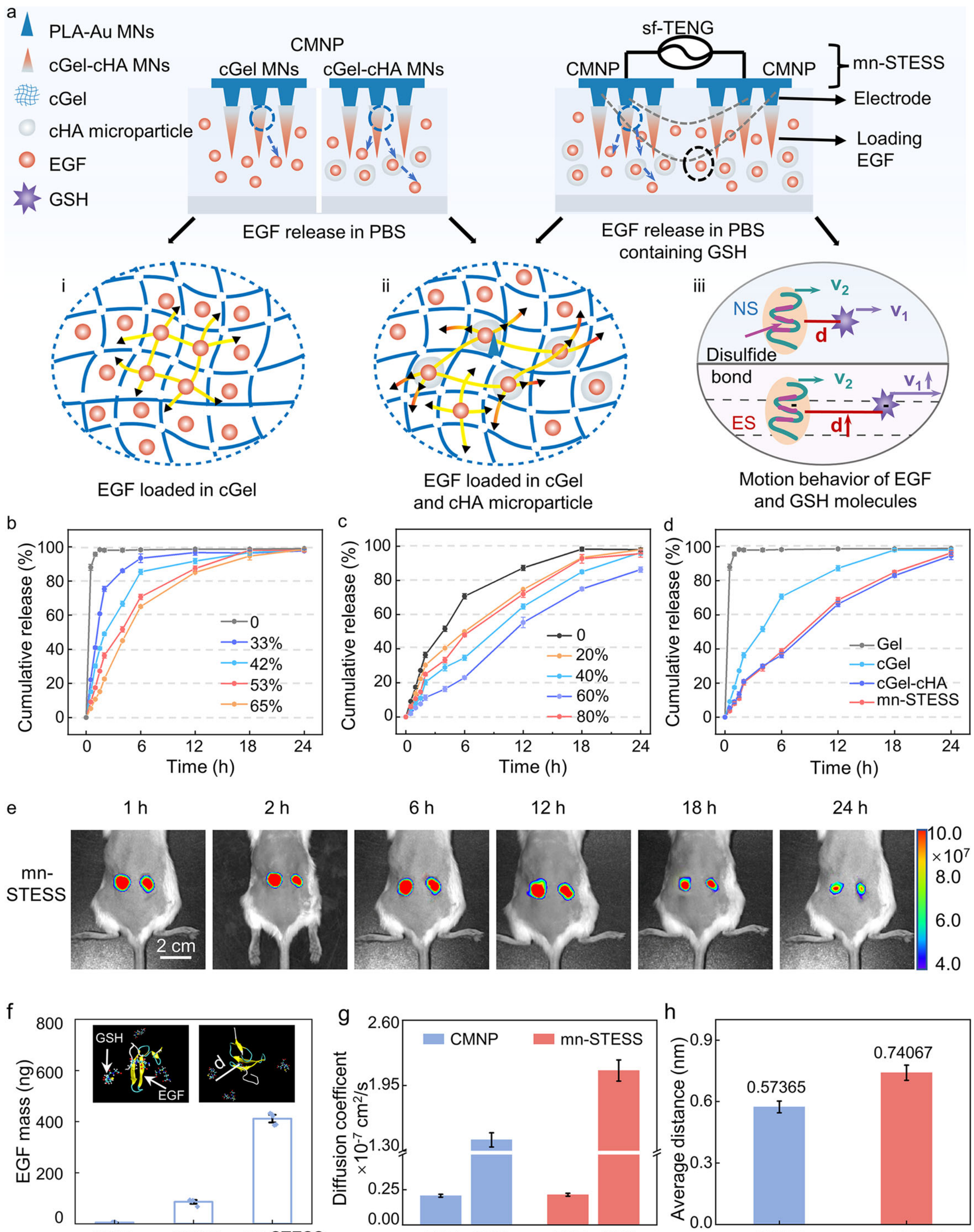

Fig. 3 | Drug delivery in vitro and in vivo. a Schematic of EGF release from MNP based on different drug-loaded materials: (i) cGel MNs; (ii) cGel-cHA MNs in CMNP and mn-STESS; (iii) motion of EGF and GSH molecules in the CMNP (NS) and mn-STESS (ES) groups. **b** EGF release efficiency from the cGel MNs with the different crosslinking degree. (*n* = 3 independent samples. Data are presented as mean ± SEM). c EGF release efficiency from cGel-cHA MNs with the different cHA microparticle contents. (*n* = 3 independent samples. Data are presented as mean ± SEM). **d** EGF release efficiency from Gel MNs, cGel MNs, cGel-cHA MNs, and mn-STESS. (*n* = 3 independent samples, mean ± SEM). **e** Fluorescence images of skin penetrated by mn-STESS at different times. Fluorescence intensities are indicated by a color scale (right). Blue to red presents the minimum to maximum fluorescence intensity. **f** Mass of EGF from CMNP and mn-STESS in GSH solution; (inset) motion behavior of EGF and GSH in the CMNP (NS, left) and mn-STESS (ES, right) groups; the yellow parts indicate the disulfide bond of EGF. (*n* = 5 independent samples. Data are presented as mean ± SEM). **g** Diffusion coefficients of EGF and GSH under NS and ES. Data are presented as mean ± SEM, and the errors are generated by the linear fit of the mean square displacement from simulation trajectory analysis. **h** Distance between EGF and GSH molecules under ES; CMNP was set as control group. Data are presented as mean ± SEM, and the errors are generated by the linear fit of the mean square displacement from simulation trajectory analysis. Source data are provided as a Source Data file.

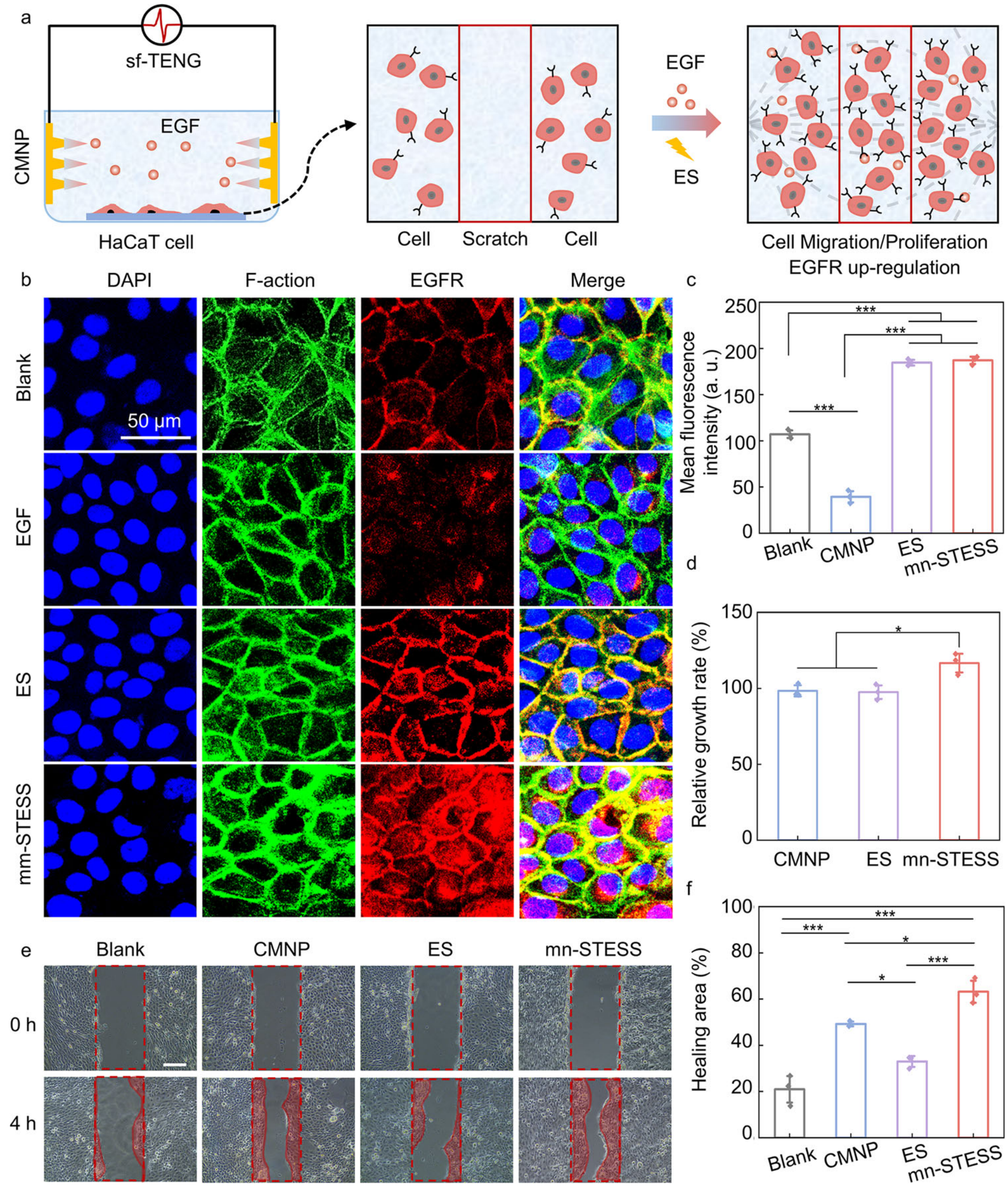

Fig. 4 | Effects of electrical stimulation synergistically with EGF on HaCaT cell behavior. a mn-STESS generated ES and released EGF to promote cell proliferation and migration. **b** Representative fluorescence images of F-action (green) and EGFR (red) in HaCaT cells treated with CMNP (EGF), ES, and mn-STESS (EGF and ES); scale bar, 50 μm. **c** Mean fluorescence intensities of EGFR expression in HaCaT cells. ($n = 3$ independent samples. ***$p < 0.001$. All statistical analyses were performed by one-way ANOVA. Data are presented as mean ± SEM). **d** Relative growth rate of HaCaT cells treated with CMNP (EGF), ES, and mn-STESS (EGF and ES). ($n = 3$ independent samples. *$p < 0.05$. All statistical analyses were performed by one-way ANOVA. Data are presented as mean ± SEM). **e** Representative images of HaCaT cell migration; red area indicated the migrated cells; scale bar, 200 μm. **f** Migration area of HaCaT cells treated with CMNP (EGF), ES, and mn-STESS (EGF and ES). ($n = 3$ independent samples. *$p < 0.05$ and ***$p < 0.001$. All statistical analyses were performed by one-way ANOVA. Data are presented as mean ± SEM). Source data are provided as a Source Data file.

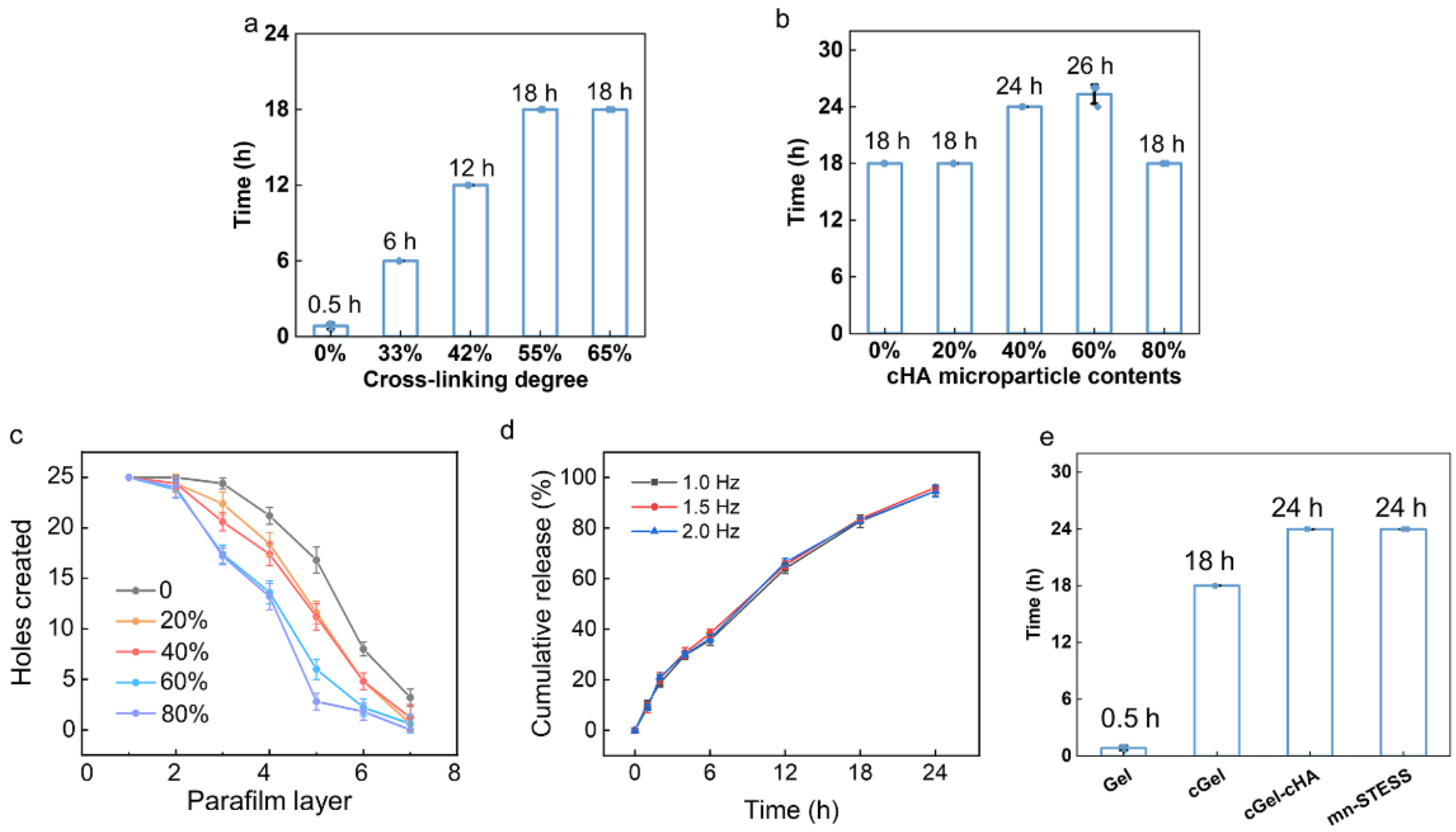

Supplementary Figure 10. EGF release rate. (a) The sustained release time of Gel MN with the different cross-linked degrees 0, 32%, 42%, 53%, 65%. (n=3 independent samples. Data are presented as mean ± SEM). (b)The sustained release time of cGel-cHA MN with the different cHA microparticle contents 0, 20%, 40%, 60%, 80%. (n=3 independent samples. Data are presented as mean ± SEM). (c) Holes created in each parafilm layer after insertion of the cGel-cHA MN with the different cHA microparticle contents. (n=5 independent samples. Data are presented as mean ± SEM). (d) EGF release efficiency from mn-STESS with different sliding frequencies. (n=3 independent samples. Data are presented as mean ± SEM). (e) The sustained release time of EGF from Gel MN, cGel MN, cGel-cHA MN and mn-STESS. (n=3 independent samples. Data are presented as mean ± SEM). Source data are provided as a Source Data file.

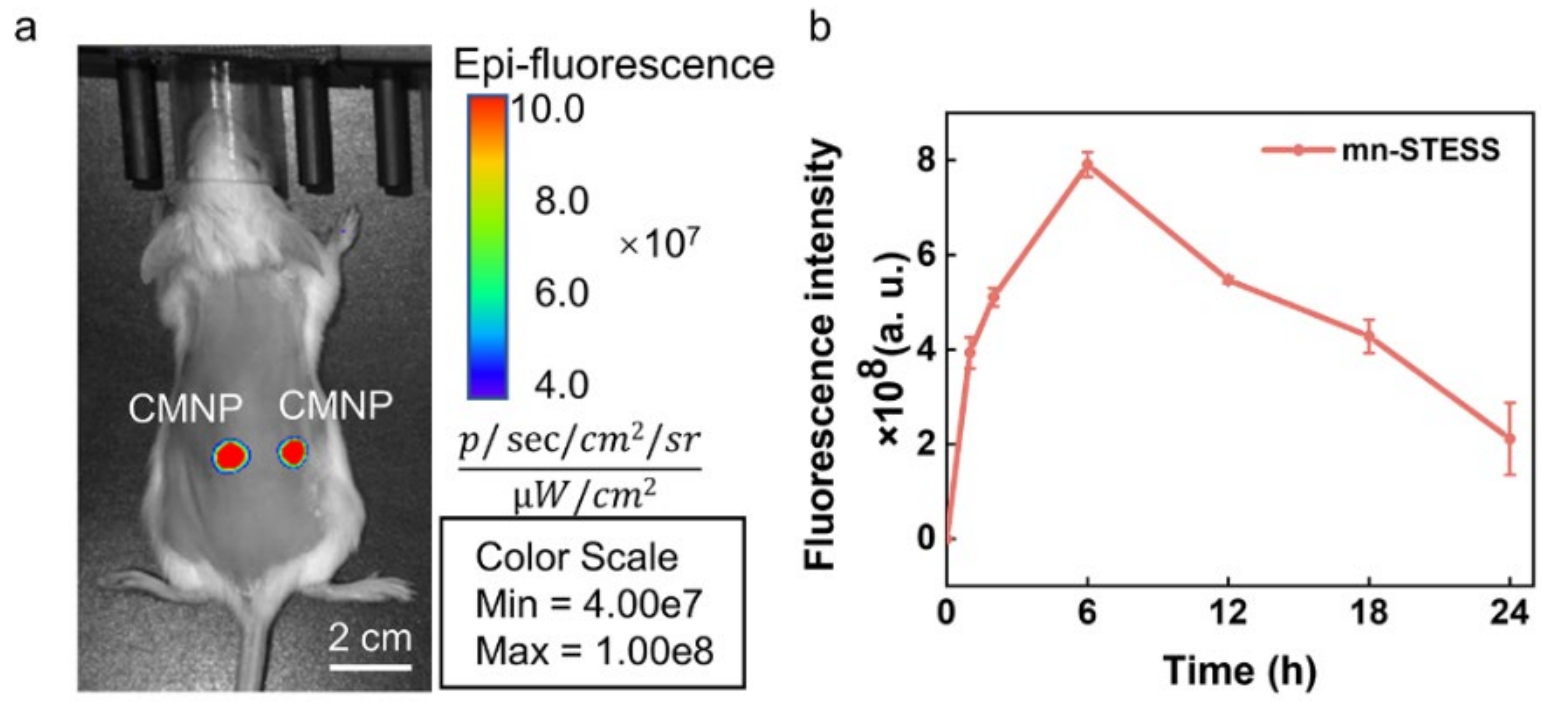

Supplementary Figure 13. Drug delivery *in vivo*. (a) Fluorescence images of skin site penetrated by CMNP. (b) Fluorescence intensity of skin site penetrated by mn-STESS at different times, CMNP as control group. (n=3 independent samples. Data are presented as mean ± SEM). Source data are provided as a Source Data file.
